# Genetic Signatures of Exceptional Longevity in Humans

**DOI:** 10.1371/journal.pone.0029848

**Published:** 2012-01-18

**Authors:** Paola Sebastiani, Nadia Solovieff, Andrew T. DeWan, Kyle M. Walsh, Annibale Puca, Stephen W. Hartley, Efthymia Melista, Stacy Andersen, Daniel A. Dworkis, Jemma B. Wilk, Richard H. Myers, Martin H. Steinberg, Monty Montano, Clinton T. Baldwin, Josephine Hoh, Thomas T. Perls

**Affiliations:** 1 Department of Biostatistics, Boston University School of Public Health, Boston, Massachusetts, United States of America; 2 Division of Chronic Disease Epidemiology, Department of Epidemiology and Public Health, Yale University School of Medicine, New Haven, Connecticut, United States of America; 3 IRCCS Multimedica, Milano, Italy; Istituto di Tecnologie Biomediche – Consiglio Nazionale delle Ricerche, Segrate, Italy; 4 Center for Human Genetics, Boston University School of Medicine, Boston, Massachusetts, United States of America; 5 Section of Geriatrics, Department of Medicine, Boston University School of Medicine and Boston Medical Center, Boston, Massachusetts, United States of America; 6 Department of Medicine, Boston University School of Medicine, Boston, Massachusetts, United States of America; 7 Department of Neurology, Boston University School of Medicine, Boston, Massachusetts, United States of America; 8 Departments of Medicine and Pediatrics, Boston University School of Medicine and Boston Medical Center, Boston, Massachusetts, United States of America; Georgia Institute of Technology, United States of America

## Abstract

Like most complex phenotypes, exceptional longevity is thought to reflect a combined influence of environmental (e.g., lifestyle choices, where we live) and genetic factors. To explore the genetic contribution, we undertook a genome-wide association study of exceptional longevity in 801 centenarians (median age at death 104 years) and 914 genetically matched healthy controls. Using these data, we built a genetic model that includes 281 single nucleotide polymorphisms (SNPs) and discriminated between cases and controls of the discovery set with 89% sensitivity and specificity, and with 58% specificity and 60% sensitivity in an independent cohort of 341 controls and 253 genetically matched nonagenarians and centenarians (median age 100 years). Consistent with the hypothesis that the genetic contribution is largest with the oldest ages, the sensitivity of the model increased in the independent cohort with older and older ages (71% to classify subjects with an age at death>102 and 85% to classify subjects with an age at death>105). For further validation, we applied the model to an additional, unmatched 60 centenarians (median age 107 years) resulting in 78% sensitivity, and 2863 unmatched controls with 61% specificity. The 281 SNPs include the SNP rs2075650 in *TOMM40/APOE* that reached irrefutable genome wide significance (posterior probability of association = 1) and replicated in the independent cohort. Removal of this SNP from the model reduced the accuracy by only 1%. Further in-silico analysis suggests that 90% of centenarians can be grouped into clusters characterized by different “genetic signatures” of varying predictive values for exceptional longevity. The correlation between 3 signatures and 3 different life spans was replicated in the combined replication sets. The different signatures may help dissect this complex phenotype into sub-phenotypes of exceptional longevity.

## Introduction

The average human lifespan in developed countries now ranges from about 80 to 85 years. Environmental factors such as lifestyle choices and where we choose to live as well as genetic factors all contribute to healthy aging. Supporting the importance of environmental factors in survival to old age is the 88 year average life expectancy of Seventh-Day Adventists [1], who by virtue of their religion have health related behaviors conducive to healthy aging.

Human twin studies suggest that only 20–30% of the variation in survival to about 85 years is determined by genetic variation [2]. However, the existence of rare families demonstrating remarkable clustering for extreme ages [3], [4], the increased relative risks of survival amongst siblings of nonagenarians [5] and of centenarians [6], [7], [8], [9], [10], [11], [12], [13], the fact that children of centenarians experience a marked delay in age-related diseases [14], and the similarity of centenarians' lifestyles to the general population [15], all argue that genetic factors play a much stronger role in living 25–35 years beyond the mid-eighties [10], [16], [17]. Impressively, siblings of centenarians born in 1900 have a relative risk of living nearly 100 years that is 8 (females) to 17 times (males) greater than that for the average of their birth cohort [10]. The rarity of the trait —only 1 centenarian amongst approximately 5,000 people in the US and only 1 supercentenarian (age 110+ years) amongst seven million people [18]— places exceptional longevity in a very different category from both average life expectancy and common complex traits associated with aging.

**Figure 1:**
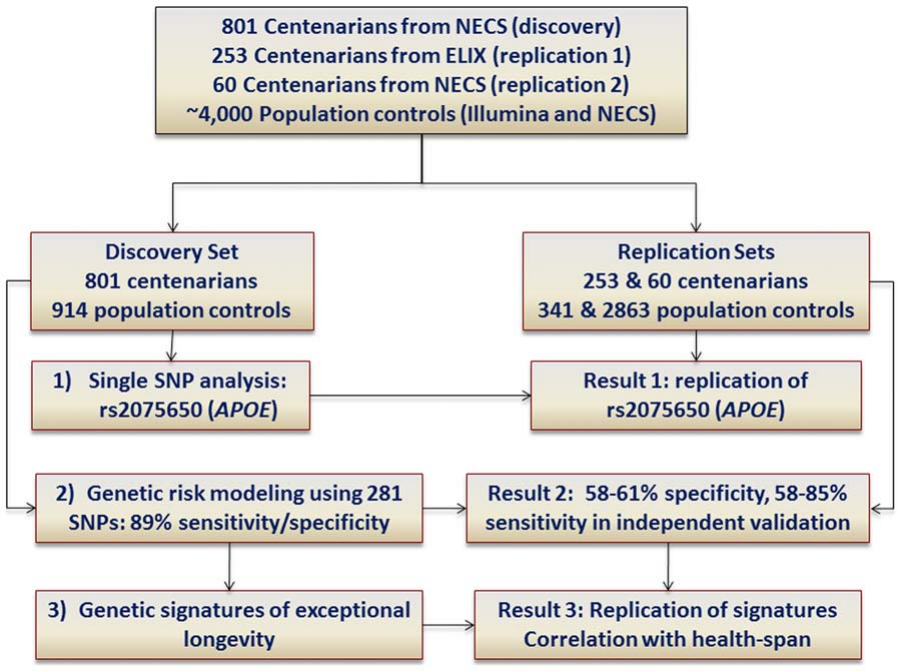
Schematic showing the methodology used to discover genetic signatures of exceptional longevity (EL). The analysis included genetic matching to remove confounding by population stratification between cases and controls of the discovery and replication set 1, discovery and replication of single SNP associations, multivariate genetic risk modeling and generation of predictive genetic profiles, and cluster analysis of genetic risk profiles to discover genetic signatures of EL.

Based upon the hypothesis that exceptionally old individuals are carriers of multiple genetic variants that influence human lifespan, we conducted a genome-wide association study (GWAS) of centenarians. We began with a traditional one SNP at a time analysis to identify SNPs that are individually associated with exceptional longevity. We then used a novel approach to build a family of genetic risk models based on Bayes rule which, while taking into account the simultaneous influence of many genetic variants, can accurately discriminate between subjects with average versus exceptional longevity. Next, we used this family of models to construct subject-specific genetic risk profiles that, by cluster analysis, can be used to discover sub-phenotypes of exceptional longevity that are characterized by different genetic signatures. **Figure 1** summarizes the steps of the analyses.

## Results

### Primary and secondary sets

**Figure 2:**
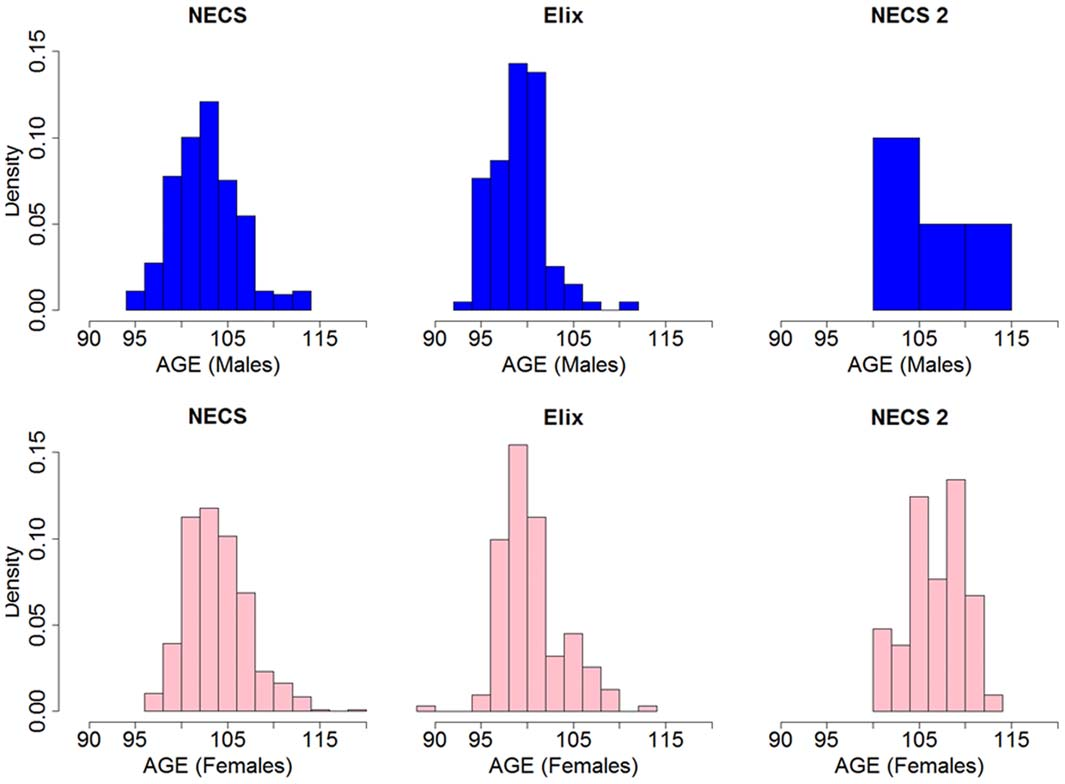
Distribution of age of last contact or age at death of centenarians included in the study. NECS: centenarians of the discovery set, ELIX: nonagenarians and centenarians from the ELIX replication set, NECS 2: additional NECS replication set of 60 centenarians. The y-axis reports the density, and the x-axis reports the age, in group of 2 years. The frequency of subjects with ages between x and x+2 is 2*density*(sample size).

Our primary set (discovery set) consisted of 801 unrelated subjects enrolled in the New England Centenarian Study (NECS) and 914 genetically matched controls. NECS subjects were Caucasians who were born between 1890 and 1910 with an age range of 95 to 119 years (median age 104 years). Approximately one-third of the NECS sample included centenarians with a first-degree relative also achieving exceptional longevity, thus enhancing the sample's power [19]. Controls included 241 genetically matched NECS referent subjects who were spouses of centenarian offspring or children of parents who died at an age ≤73 years, and 673 genetically matched subjects selected from the Illumina control database. For genetic matching we used a previously described algorithm [20] that groups subjects by ethnicities based on cluster analysis of the most informative principal components of genome-wide genotype data (**Figure S1**). Note that, based on the U.S. Social Security Administration's 1920 birth cohort life table, the average life expectancy in the cohort is 82 years, with standard deviation of 7.9 years, so that the mean age of the cases in our study and the average life expectancy in the cohort differ by 2.69 times the standard deviation. Furthermore, the mean age of NECS controls was 75 years, with standard deviation 7 years. Therefore, the difference between mean age of centenarians in the discovery set and NECS controls was more than 4 times the standard deviation, thus boosting the power of the study. For replication we used two additional sets. The replication set 1 (“ELIX”) consisted of 253 North American Caucasian subjects enrolled by Elixir Pharmaceuticals between 2001 and 2003. These individuals were born between 1890 and 1910 (age range of 89–114 years, median age 100) and were recruited and phenotyped using a protocol similar to the NECS. Referent subjects (n = 341) were identified from the remaining Illumina controls and genetically matched to the 253 cases using the same matching algorithm used in the discovery set. The replication set 2 was composed of 60 centenarians that included 39 subjects of European ancestry enrolled in the NECS between June 2009 and September 2010 (age range 100–114, mean age 108) plus 21 centenarians (age range 101–115, mean age 107) not included in the discovery set during the genetic matching, and all available Caucasians samples from the Illumina control database not used in the above comparisons. Centenarians and controls in replication set 2 were not genetically matched to test the generalizability of the results. **Figure 2** displays the age distributions of centenarians in the discovery and replication sets 1 and 2. We also used an additional set of 867 neurologically normal subjects used as controls for a Parkinson's disease GWAS [21], to test the robustness of single SNP associations. We analyzed 243,980 SNPs that passed a stringent quality control protocol described in the methods.

### Single SNP Analysis

First we conducted a traditional single SNP analysis in which we ranked SNPs in the discovery set by the strength of association. We employed both Bayesian and traditional frequentist analyses of 4 different genetic models (general/genotypic, allelic/additive, recessive and dominant associations) to maximize power [22], [23]. With the Bayesian analysis, we scored each SNP association by the Bayes Factor (BF), which is the posterior odds for the association when the null hypothesis of no association and the alternative hypothesis of an association have the same prior probability [24], and then we used the maximum BF (MBF) as a measure of statistical significance. **Figure S2** shows the error rate of decision rules based on several thresholds for MBF. The matching strategy appeared to remove confounding by stratification because we did not observe any inflation of associations and the genomic control factor in allelic association was 0.99 (**Figure S3**). We also conducted additional analyses described below to investigate whether residual confounding by population stratification could bias the results and found no evidence of bias.

**Figure 3:**
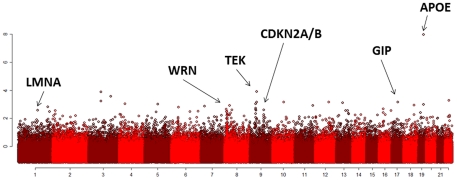
The Manhattan plot displays the maximum log10(Bayes Factor) (y-axis) for each of the analyzed SNPs in the discovery set. The Manhattan plot displays the maximum log10(Bayes Factor) (y-axis) for each of the analyzed SNPs in the discovery set. The SNPs are ordered by chromosome (alternate color bands) and, within chromosome, by physical position (x-axis). We tested the association of each SNP with exceptional longevity using general, allelic, dominant and recessive models and the y-axis reports the maximum log10(Bayes factor) observed for each SNP. The SNP rs2075650 in *APOE/TOMM40* reached irrefutable genome wide significance (log10(MBF) = 7.9 and p-value<e-10). Figure S3 shows the Manhattan plot and QQ plot for the additive model using logistic regression.

The Manhattan plot (**Figure 3**) displays the log10(MBF) for each tested SNP. This analysis identified a single SNP in *APOE/TOMM40* as irrefutably genome-wide significant (P<10e-8, **Table 1**). The association was replicated in the ELIX set, and was maintained when we used 867 referent subjects included in a GWAS of Parkinson's disease as alternative controls (**Table 1**).

**Table 1:** Replication of the association of rs207650 in TOMM40/APOE.

	SNP	Gene	Chrom	Alleles	Discovery Set (801, 914)			
					LOG10(BF)	p-value	OR	p(A)
Discovery Set (801, 914)	rs2075650	***TOMM40/APOE***	chr19:50087459	AG/GG v AA	6.31	1.03E-08	0.49	0.15/0.26
Replication Set (Elix 253, 341)					2.04	0.000468	0.47	0.15/0.27
Combined (1054, 1255)					9.30	1.01E-11	0.48	0.15/0.26
Coriell (801, 867)					3.73	3.86E-06	0.55	0.15/0.24

The table shows the replicated associations of the SNP rs207650 in *TOMM40/APOE* in the replication set 1 and the additional control set from the Parkinson's Disease study. Column legends: **SNP** = official dbSNP identifier. **Gene** = official gene name for SNPs that are within 20 kb from transcribed regions. **Chrom** = Chromosome and physical position of SNP in hg18. **Alleles** = the two SNP alleles (allele 1 v allele 2) in the genetic model that reached strongest significance in the Bayesian analysis. **LOG10(BF)** = the logarithm 10 Bayes Factor for the association relative to the null model of no association. Assuming uniform prior probabilities for the two hypotheses, the BF represents the posterior odds for association. **P-value** = p-value for 1 degree of freedom test for the dominant model AG/GG versus AA. **OR** = odds ratio for exceptional longevity in subjects who carry allele 1 relative to allele 2. For example, subjects who carry the allele 1 (AG/GG) of SNP rs2075650 have 0.49 times the odds for exceptional longevity compared to subjects who carry the allele 2 (AG/GG: either the genotype AG or GG). **P(A)** = prevalence of allele 1 in cases and controls. For example, 15% of centenarians carry the allele AG/GG of SNP rs2075650 compared to 26% of controls. Row 1 shows the results in the discovery set; row 2 in the ELIX set, row 3 the combined discovery and ELIX datasets and row 4 is the set in which the 914 matched controls of the discovery set were replaced with the unmatched Coriel controls.

The apolipoprotein E (*APOE*) is associated with human lifespan [25], [26], [27]. SNP rs2075650 occurs in an intron of *TOMM40* but it is a strong proxy of the SNPs that define the *APOE* alleles [28]. This SNP has been associated with Alzheimer's disease (AD) [29], [30] and lipid levels [31], [32].

### Genetic Risk Modeling

In the single SNP analysis, we observed a substantial enrichment for significant associations which do not meet the stringent threshold for genome wide significance. For example, 112 SNPs were associated with exceptional longevity with log10(MBF)>2 against an estimated error rate of 4 in 100,000 independent tests and hence 8–10 false positive associations expected by chance in ∼250,000 tested SNPs if there were no significant associations and all SNPs were independent (**Figure S2**). The clusters of associations in chromosomes 8, 9 and 21 in **Figure 3** point to interesting regions, although they fail to reach genome wide significance. Several authors have argued that SNPs that do not reach genome wide significance may be biologically important by virtue of their joint effect [33], [34], [35], [36], and have successfully built risk models that can predict genetic susceptibility to several complex traits that are highly heritable [37], [38], [39], [40], [41]. We similarly explored the hypothesis that different sets of SNPs that are associated with exceptional longevity, although with moderate effects, may jointly characterize the genetic predisposition to exceptional longevity [42], [43] and therefore provide a model for in silico analysis that can suggest targets and genetic paths to exceptional longevity.

#### Selection of Predictive SNPs

**Figure 4:**
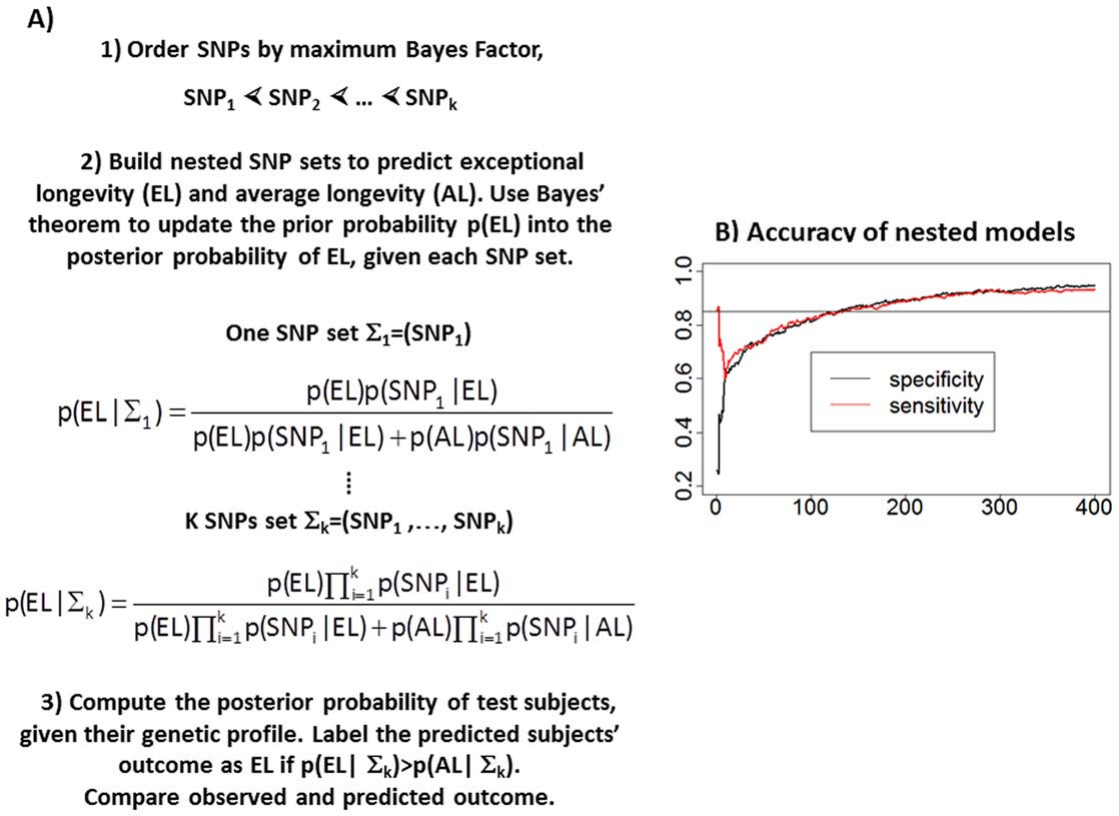
A) Schematic illustration of the genetic risk prediction model. We ordered SNPs by maximum Bayes Factor in the discovery set and built nested SNP sets starting with the most significant SNP and then adding one SNP at a time from the ordered list. The conditional probabilities of SNP genotypes in centenarians (p(SNP_i_|EL)) and controls (p(SNP_i_|AL)) are used to compute the posterior probability of exceptional longevity (p(EL|Σ_k_)) using Bayes' theorem and prior probability p(EL) = 0.5. The classification rule is the standard Bayesian classification rule that is optimal under a 0–1 loss function. **B) Sensitivity and specificity of 400 nested models.** The x-axis reports the number of SNPs in each of the nested models, and the y-axis reports sensitivity (% of centenarians with posterior probability of exceptional longevity>posterior probability of average longevity) and specificity (% of controls with posterior probability of exceptional longevity<posterior probability of average longevity).

To proceed with this analysis, we had to make several decisions about the class of models to work with, how to determine the number of SNPs to be included in the model, and the overall search strategy. We chose to compute the genetic risk associated with a set of SNPs using a simple but effective Bayesian classification model, also known as the naïve Bayes classifier (**Figure 4A**) [44]. This approach –also used in [39] to accurately predict the susceptibility to carotid atherosclerosis – classifies a subject as predisposed to exceptional longevity if the posterior probability of exceptional longevity, given genotypes of a set of SNPs, exceeds the posterior probability of average longevity (**Figure 4A**). The advantage of this method is that there is virtually no upper limit to the number of SNPs that can be used for classification, and it can be used for risk prediction even if the data used for the analysis are from a case control study. We designed a forward search procedure to discover a sufficient number of predictive SNPs (**Figure 4A**). The procedure builds a series of nested genetic risk models starting with the most significant SNP in the discovery set and incrementally adding one SNP at a time from a pruned set of SNPs that are sorted in order of log10(MBF). Each model is used for prediction, and the accuracy of each model to predict exceptional longevity and average longevity is evaluated by sensitivity and specificity (**Figure 4B**). The trend of sensitivity and specificity in **Figure 4B** shows that including more SNPs increases both sensitivity and specificity but the gain of accuracy becomes less and less as SNPs with decreasing statistical significance (lower MBF) are added. Particularly, the sensitivity plateaus between 275–285 SNPs so that including more SNPs does not appear to improve the sensitivity further (**Figure 4B**). Because the model with 281 gives the closest sensitivity and specificity, we stopped the search for predictive SNPs at 281. We also used a resampling approach (**Figure S4A**) to validate this choice, and examined the effect of changing the SNP order in our heuristic search (**Figure S4C and D**), and possible lab-genotyping bias (**Figure S4B**).

**Table S1** provides complete details of all of the 281 SNPs, and the probabilities that are used to compute the prediction using the formula in **Figure 4A**. Reliability of the Illumina genotyping was double-checked by re-genotyping the top 28 SNPs of the model using TaqMan genotyping in an independent lab, and the 99.7% concordance suggests that the data are reliable (**Figure S5**). Intensity plots of the 281 SNPs are available from www.bumc.bu.edu/centenarian. 137 SNPs of the 281 SNPs occur in 130 genes, some of which have been previously associated with aging such as *LMNA* (rs915179), *WRN* (rs1800392), and *SOD2* (rs2758331) and several of them are in close proximity of coding SNPs [45]. The *LMNA* gene, which encodes the nuclear envelope proteins lamin A and lamin C, has been associated with the progeroid (premature aging-like) syndrome, Hutchinson-Gilford syndrome [46]. The *WRN* gene is a DNA helicase and exonuclease that plays a deterministic role in DNA repair and another progeroid syndrome, Werner's Syndrome [47]. The *WRN* gene has been associated with longevity in the Framingham Heart Study (FHS) sample [48]. It is remarkable that the two genes responsible for the best known progeroid syndromes appear in the genetic risk model, and this may reflect the power of the discovery sample which includes such extreme old ages. Another gene, also noted to be associated with longevity in the FHS sample as well as the Jerusalem Study, is *SOD2*, or superoxide dismutase 2 [49]. *SOD2* is a key free radical scavenger and free radical damage likely plays an important pathogenic role in aging and numerous age-related diseases [50]. *CDKN2A* (rs1063192) performs a key step in the p53 pathway that has been posited to play a key role in inducing cellular senescence [51] and it has been associated with adult onset diabetes [52]. *SORCS1* (rs7907713) and *SORCS2* (rs6812745) have been linked AD [53]. Gastric inhibitory polypeptide (*GIP*), commonly referred to as glucose-dependent insulinotropic peptide, encodes a protein that regulates insulin secretion and activates *AKT* [54]. The association of this gene (rs9899404) supports the potential role of insulin regulation in exceptional longevity [55], and suggests new target genes for human aging beyond *FOXO1*, *FOXO3A* and *IGF-IR* [56], [57], [58]. There is also growing evidence of *GIP* playing a protective role in both diabetes and AD and GIP is being investigated as a therapeutic target [59].

**Figure 5:**
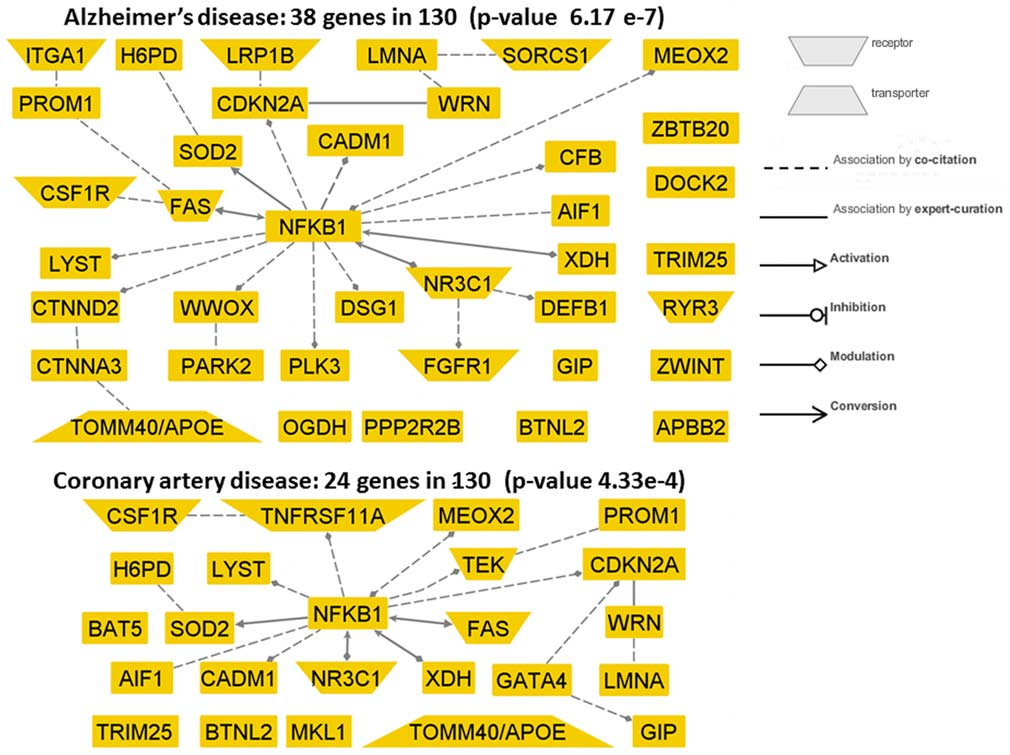
Genes in the genetic risk models have been linked to coronary artery disease and Alzheimer's disease. The two networks display 38 of the 130 genes in the genetic risk model that are linked to Alzheimer's disease (top) and 24 of the 130 genes that are linked to coronary artery disease (bottom) in the literature, either by functional or genetic association studies. The nodes that are linked by an edge represents either genes that are “co-cited” (dashed lines) or “associated by expert curation” (continuous lines). The arrow head means that the associations are activation (triangle), inhibition (circle), modulation (diamond), conversion (arrow head). The node shape informs about known roles of the genes (see inset). The nodes that are singleton were linked to AD/CAD in the literature but not together with other genes. The number of genes linked to each disease was compared to what is expected by chance using Fisher exact test, and the p-values show that the gene seta are unluckily the result of chance. (Networks generated with Genomatix).

We used Genomatix (http://www.genomatix.de) to annotate the list of 130 genes included in the genetic risk model and the analysis showed that the list was enriched for several groups of genes linked to both common and rare diseases (MeSH). Genes related to Alzheimer's disease, dementia and tauopathies were the most significant: 38 of the 130 genes were linked to AD in the literature (p-value to test the null hypothesis that this happens by chance was **6.17 e-7**) and they are displayed in **Figure 5**; 42 genes were linked to dementia (**Figure S6**, p-value to test the null hypothesis that this happens by chance was **1.07 e-6**) and 38 to tauopathies (p-value 8.47e-7). The fact that so many genes are noted to play a role in dementia is consistent with the epidemiologic finding that dementia is absent or markedly delayed amongst centenarians (average age of onset, 93 years) [60]. Genes related to other age related diseases were also significantly represented: 24 genes were linked to coronary artery disease (**Figure 5**), and several genes were linked to neoplasms.

#### Genetic Risk Profiles and Ensemble of Risk Models

**Figure 6:**
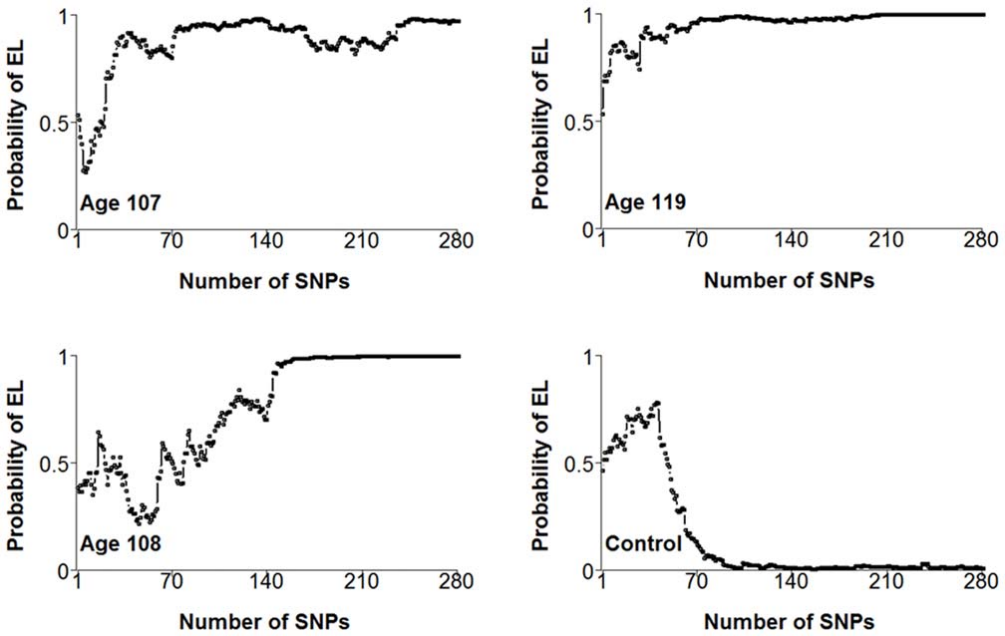
Examples of genetic risk profiles in 4 study subjects (3 centenarians with ages at death 107, 108 and 119 years, and a control). 281 nested SNP sets were used to compute the posterior probability of exceptional longevity in the 4 subjects (y-axis) and were plotted against the number of SNPs in each set (x-axis). In the 107 year old, the first 5 SNP sets Σ_1_ = [rs2075650], Σ_2_ = [Σ_1_, rs1322048], …, Σ_5_ = [Σ_4_, rs6801173] determine a posterior probability of exceptional longevity ranging between 0.54 and 0.28. This subject carries genotypes AA, AG, AG, CC, AA for the 5 SNPs respectively and, with the exclusion of genotype AA of rs2075650 that is more common in centenarians, the other genotypes are more common in controls than centenarians and determine a posterior probability of exceptional longevity that is lower than the posterior probability of average longevity. The sixth SNP set, Σ_6_ = [Σ_5_, rs337656], predicts an almost 30% chance of exceptional longevity. The subject carries the AA genotype for the SNP rs337656 that is more frequent in centenarians (Table S1), and carrying this genotype increases the posterior probability of exceptional longevity. The probability predicted by the next SNP sets increases steadily and all models with more than 20 SNPs predict more than a 50% chance of exceptional longevity. This genetic profile shows that the subject carries some combinations of SNP alleles that are associated with exceptional longevity, while other alleles are associated with “average longevity”. However, the overall genetic risk profile determined by all 281 SNP sets makes a strong case for exceptional longevity because the majority of models predict more than an 80% chance of exceptional longevity. The genetic risk profile of the centenarian who died at age 119 years is even more convincing: with the exception of the first SNP, all subsequent SNP sets determine more than a 70% chance of exceptional longevity, and 272 of the 281 models predict more than an 80% chance for exceptional longevity. This profile shows that this subject is highly enriched for SNPs alleles that are more common in centenarians (longevity associated variants) and that probably played a determinant role in the extreme survival. The profile of the third subject, age 108 years, shows that different SNP sets determine different chances for exceptional longevity, and only the overall trend of genetic risk provides evidence for exceptional longevity. The fourth plot displays the profile of a control, and shows that this subject carries some longevity associated variants; however, the overall trend of genetic risk points to average longevity rather than exceptional longevity.

To better understand the role of these 281 SNPs in shaping the genetic susceptibility to exceptional longevity, we generated a genetic risk profile for each subject by plotting the posterior probability of exceptional longevity (p(EL|Σ_k_), y axis) against the number of SNPs in each of the 281 SNP sets Σ_k_ (x-axis) and examined their patterns. **Figure 6** shows, for example, the profiles from 3 centenarians and a control. In each profile, an increasing posterior probability of exceptional longevity shows strong enrichment of longevity associated variants, because the posterior probability of exceptional longevity increases when the profile includes a new SNP genotype that is more frequent in centenarians than in controls (see methods).

**Figure 7:**
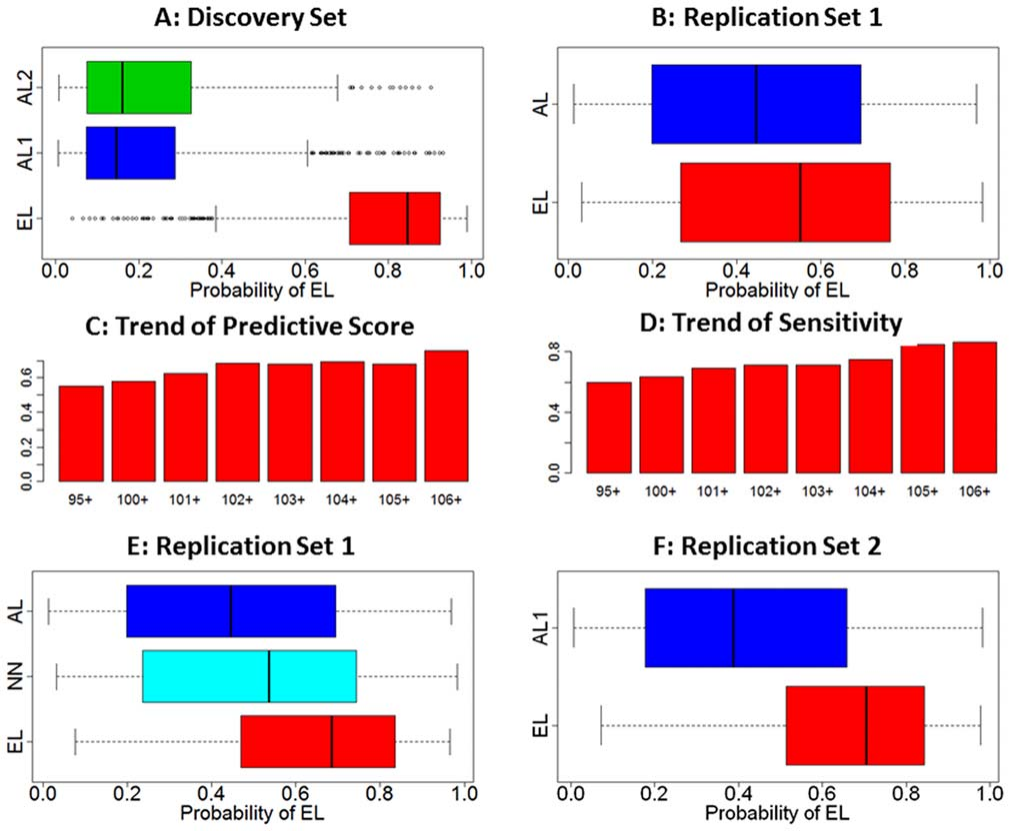
Discrimination of the classification rule based on the ensemble of 281 genetic risk models. **Panel A:** Posterior probability of exceptional longevity (EL) and average longevity (AL) (x axis) in the centenarians (red boxplots) and controls (AL1: Illumina controls, blue boxplots, AL2: NECS controls, green boxplots) of the discovery set (NECS, top left). Both sensitivity and specificity were 89%. The boxplots in blue and green show that the distributions of the posterior probability of EL in the two control groups are not statistically different (p-value from t-test comparing the posterior probability of EL = 0.21). **Panel B:** Posterior probability of EL and AL (x axis) in the centenarians (red boxplots) and controls of the replication set 1. Sensitivity and specificity were 60% and 58% and the distributions of the predictive score are significantly different (t-test p-value = 0.001). **Panel C:** Median values of the posterior probability of EL (predictive score) in subsets of centenarians of the replication set 1 with increasing ages. The barplot shows that the median score increases with older ages. **Panel D: Sensitivity of the classification rule in subsets of centenarians of the replication set 1 with increasing ages.** The barplot shows the increasing sensitivity in older groups that reaches 85% in 20 subjects aged 106 and older. **Panel E: Distribution of the posterior probability of exceptional longevity in the 253 cases of the replication set divided into two age groups (<103 years, pale blue, mean age 99 years, and ≥103 years, red, mean age 106).** The sensitivities in the two groups are 57% and 71.4%. The three distributions are significantly different (p-value = 0.04 from t-test comparing Illumina controls and centenarians aged <103; p-value = 0.004 from t-test comparing the centenarians stratified by age). **Panel F: Sensitivity and specificity in an additional set of 2863 controls from the Illumina database (blue), and an additional set of 60 centenarians that include 39 centenarians enrolled since June 2009 (mean age 108) and 21 centenarians that were excluded from older analysis because of genetic matching (mean age 106).** The specificity in the additional Illumina controls is 61.2%. The sensitivity in the additional centenarians was 71.5% in the set of 21, and 82% in the additional 39 for a total of 78% (p-value from t-test comparing the posterior probabilities of EL in controls and centenarians <1e-10).

These examples support the hypothesis that exceptional longevity is determined by varying combinations of longevity associated variants and some number of SNPs may be optimal for classifying some subjects but not others. Consistent with this observation, we choose an ensemble of all 281 genetic risk models to compute the posterior probability of exceptional longevity. This ensemble of 281 genetic risk models provides 89% specificity and sensitivity in the discovery set (**Figure 7A**). We next evaluated the predictive accuracy of this ensemble of models in the two replication sets, the ELIX set and a recently enrolled sample of NECS centenarians.

Sensitivity and specificity in the replication set 1 (the ELIX sample) comprised of 253 nonagenarians and centenarians and 341 genetically matched controls were 60% and 58% (**Figure 7B**) and AUC = 0.58 (**Figure S7**). Although the distributions of the predictive scores are significantly different (p-value from t-test comparing the predicted probabilities of exceptional longevity in the two groups was 0.001), the discrimination of the model is less remarkable. Since the ages of subjects in this replication set are younger compared to the centenarians in the discovery set (median age in the ELIX set was 100 years compared to 104 in centenarians of the discovery set) and because we expect that the genetic component of exceptional longevity increases with age, we next examined the distribution of the predictive score and the trend of sensitivity in subsets of subjects with older ages. The median probability of exceptional longevity in subsets of increasing age of survival increases to more than 68% in the 81 subjects with ages >101 (**Figure 7C**) and, consistently, the sensitivity of the model to correctly classify older subjects increases with older ages and reaches 85% in 20 subjects ages 106 and older (**Figure 7D**). For example, when the 253 cases of the replication set were divided into two age groups to better match the ages of the substantially older discovery set (204 subjects, age <103, median age 100 years, and 49 subjects, age ≥103, median age 105) the sensitivity of the model was 71% (**Figure 7E**).

To further investigate our hypothesis that the genetic contribution to exceptional longevity increases with older ages we evaluated the sensitivity of the classification rule in a second replication set of newly enrolled NECS centenarians (n = 39) plus NECS centenarians not included in the discovery set (n = 21), the sum of which had a median age of 107 years (**Figure 7F**). The sensitivity was 78% (71.5% in the group of 21 with median age 106 and 82% in the recently enrolled and older group of 39) confirming increasing sensitivity with increasing ages. The boxplot in **Figure 7F** shows that the specificity in an additional set of 2863 controls of replication set 2 was is 61.2%, and the AUC in this second replication set was 0.74 (**Figure S7**). **Figure S8** shows that classification rules based on randomly ordering the top 281 SNPs (mid panels) or selecting 281 SNPs at random have lower sensitivity and specificity.

Our analysis used genetic matching to remove confounding by population structure. However, since we matched subjects within clusters, residual stratification might still confound the association and possibly affect the classification rule. To test the hypothesis that there is no confounding by residual stratification, we conducted two traditional analyses. In one analysis, we adjusted the associations of the 281 SNPs by the top 4 principal components, and in the second analysis we did not. We then checked whether adjusting the analysis by the principal components would change the results of the unadjusted analysis. **Figure S9** shows that the distributions of p-values for the two analyses in different genetic models are essentially identical (correlation coefficient 0.98 to 0.99). This analysis would indicate that there is no confounding due to residual stratification. We repeated the analysis adjusting for the top 10 principal components. The effect of this more stringent adjustment made 3 of the 281 SNPs borderline significant. We also checked if there is any residual correlation between the top two PCs and the score predicted by our model, and there appears to be none (**Figure S10**).

### Genetic Signatures

Some genetic risk profiles were recurrent and we speculated that groups of centenarians may have genetic risk profiles that are associated with different sub-types of exceptional longevity such as different prevalences or ages of onset of age-related diseases. To test this hypothesis, we used cluster analysis to group the genetic risk profiles into prototypical signatures. We then investigated whether groups of centenarians with particular genetic risk profiles shared specific age-related sub-phenotypes.

**Figure 8:**
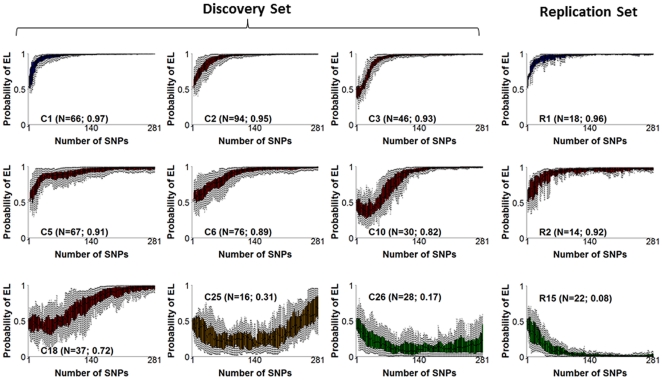
Example of 9 clusters of genetic risk profiles in centenarians of the discovery set and 3 similar clusters in replication sets 1 and 2. In each plot, the x-axis reports the number of SNPs in each genetic risk model (1,…,281), and the y-axis reports the posterior probability of exceptional longevity predicted by each model. The boxplots (one for each SNP set on the x axis) display the genetic risk profiles of the centenarians grouped in the same cluster. Numbers N in parentheses are the cluster sizes, and the average posterior probability of exceptional longevity. Color coding represents the strength of the genetic risk to predict EL (Blue: P(EL|∑_281_)>0.95; Red: 0.5<P(EL|∑_281_)<0.95; Orange: 0.20<P(EL|∑_281_)<0.5; Green: P(EL|∑_281_)<0.2). The full set of 26 clusters is in **Figure S11** and includes more than 90% of centenarians in the discovery set.

Cluster analysis identified 26 groups of 8 to 94 centenarians (90% of the discovery set) with similar genetic risk profiles, while 10% of the centenarians had rare profiles that occur in groups of 7 centenarians or less. **Figure 8** shows, for example, the 9 largest clusters while all clusters are shown in **Figure S11**. The prototypical genetic risk profiles associated with each cluster are informative displays of the longevity associated variants, and represent different genetic signatures of exceptional longevity. While the ensemble of genetic risk models provides a global estimate of the probability of exceptional longevity, the pattern itself provides information about the different sets of longevity associated variants that drive a subject toward this probability. The same cluster analysis of predicted profiles in centenarians of the merged replication sets 1 and 2 identified 15 clusters with 8 or more subjects, while approximately 35% profiles clustered in groups of 7 or less. The two most predictive and the one least predictive clusters from the replication set are also shown in **Figure 8** **. Figure S12** depicts all 15 clusters with 8 or more subjects in the merged replication sets.

To examine the specificity of the profiles in characterizing exceptional longevity, we also generated genetic risk profiles of the control subjects in the discovery set and used cluster analysis to group them. Only 5 subjects had profiles that predicted exceptional longevity with more than 90% posterior probability (**Figure S13**). Other clusters with more than 8 subjects show that the majority of these profiles match either the lack of a predictive genetic signature as in cluster C26 or the sporadic presence of longevity associated variants of clusters C24–C25 in **Figure S11**. To further extend this analysis, we clustered the genetic profiles of all 4118 controls that include all controls in the discovery and replication sets 1 and 2. Cluster analysis identified several signatures, of which only 17% predict exceptional longevity with more than 70% posterior probability, and 67% predict average longevity (**Figure S14**). The most predictive genetic signatures that characterize exceptional longevity are rare amongst control subjects, and only 0.6% of the genetic signatures of control subjects have a posterior probability of exceptional longevity >0.95.

Interestingly, the patterns of genetic risk profiles that cluster into genetic signatures distinctly differ from clusters of genetic risk profiles generated from SNPs selected at random (**Figure S15**). We also investigated if some clusters were enriched for specific ethnicities, but no clusters showed enrichment for any specific European ethnicity.

**Figure 9:**
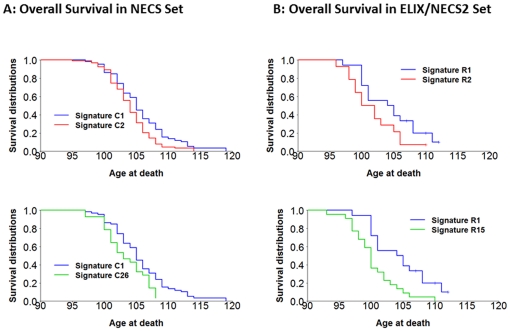
Correlation of genetic signatures with lifespan. **Panel A:** Some genetic signatures are associated with significantly different life-span. For example the most predictive signature (C1) comprises centenarians with significant longer survival compared to centenarians with signatures C2 or C26. (p-value 0.01 and 0.02) More examples are in **Figure S15**. **Panel B:** The two most predictive genetic signatures and the least predictive signature in the centenarians of the merged replications sets show consistent results. The comparison between survival of centenarians with the most predictive signature R1 and the least predictive signature R15 reaches statistical significance, (p-value = 0.003) while the comparison between survival distributions of centenarians with signatures R1 and R2 does not reach statistical significance (p-value 0.10).

We next investigated whether different genetic signatures correlate with different life spans (**Figure 9**). Some genetic signatures were indeed associated with significantly different life spans. For example, the most predictive signature (C1) was comprised of centenarians with significantly longer survival compared to centenarians with signatures C2 (the second most predictive) or cluster C26 (the least predictive), and the median survival in centenarians with signature C1 was 105 years compared to 104 years in centenarians with signature C2 or 103 years in centenarians with signature C26. We observed a similar result when we compared the survival of centenarians with the most predictive signatures in the merged replication sets (R1 and R2), and when we compared the survival of centenarians with the most and the least predictive signatures (R1 and R15) (See **Figure 9**). However, not all signatures correlated with different survival, for example centenarians with signatures C1 and C3 did not demonstrate different survival (See **Figure S16**). Preliminary analyses provided in the supplementary material (in need of replication) suggest that the different genetic signatures of exceptional longevity associate with varying prevalences and ages of onset of various age-related diseases (**Figure S17**, **Table S2**).

For 17 of the 28 centenarians in cluster C26 who lack almost all the longevity associated variants discovered in this study, we had information about familial longevity. Twenty-five percent (n = 5) had >50% of siblings who survived past the age of 90 and some had evidence for longevity as shown in some pedigrees in **Figure S18**. This could indicate that such families have more private or rare variants not captured by either the genotyping or the model.

## Discussion

Though living to very old age runs strongly in families, it is also a very complex phenomenon with many different patterns of survival that include disease-free survival but also survival with various age-related diseases. Given this complexity, it is extremely unlikely that a single or few genes confer this survival advantage, but rather it is likely that many genes are involved. To capture this genetic complexity we developed an approach that uses genetic risk modeling for in-silico genetics. Our approach includes 3 steps: 1) a single SNP analysis to identify and rank SNPs that are significantly associated with exceptional longevity, 2) genetic risk modeling based on nested Bayesian classifiers that produce genetic risk profiles and 3) cluster analysis of the profiles to discover genetic signatures and correlate these to different survival patterns or subphenotypes of exceptional longevity.

### Limitations

Although we elected to work with naïve Bayesian classifiers, many alternative approaches to genetic risk modeling exist and our method could be extended and/or improved to include for example different parametric models, or different types of cluster analyses to discover genetic signatures. We conducted extensive simulation studies to compare our approach to logistic regression that use the genetic data, or a summary of the genetic data in a genetic risk score. Our analyses show that when all SNPs have an additive effect, using a Bayesian classifier or a logistic regression model with a weighted genetic risk score perform equivalently. However, when the genetic effects include different models of inheritance, such as a combination of dominant/recessive/general associations, then a Bayesian classifier is more robust than logistic regression with a weighed genetic risk score. In either case, the approach we chose guarantees robustness as indicated in simulation studies (Clustering by genetics ancestry using genome-wide single nucleotide polymorphisms and incorporating genetic ancestry into genetic prediction models, Doctoral dissertation by Nadia Solovieff, May 2011, available upon request). Furthermore, many other “machine-learning type” approaches exist that can be used to generate genetic risk models, and years of comparative evaluations in the machine learning community have shown that there is no clear winner, but different problems require different solutions [61]. In our search for genetic predictors of exceptional longevity, Bayesian classifiers appear to perform reasonably well and can be extended to more general directed graphical models to include interactions between SNPs and between genes and environmental factors [62]. Our approach for selecting predictive features appears to work well in this application. However other search procedures for feature selection need to be explored and may produce even better predictive accuracy.

There are aspects of our method that are based on heuristics. For example, our choice of the number of SNPs to be used in the genetic risk modeling is based on a heuristic rule. The choice of the optimal number of features to be used in a classifier is a well-known problem, with no simple solution [44] and to limit the effect of a sub-optimal selection we used an ensemble of classifiers to gain robustness. This approach is known to produce better classifiers than one single model [63]. Our heuristic search orders SNPs by maximum Bayes factor. Our secondary analyses show that random reordering of the 281 SNPs decreases the specificity slightly and selecting SNPs at random from the most significant 1700 SNPs gives models that are less predictive in independent sets (**Figure S4 and S8**). If other investigators apply this approach to other domains, they may want to conduct similar secondary analyses to evaluate whether the same heuristics lead to better models.

A major challenge we faced with our genome-wide association study was the choice of appropriate controls. Because of the limited number of controls in the NECS, we had to resort to healthy controls from other genome-wide association studies (the Illumina control data set and the NECS controls where genotype data were generated in different labs with different SNP arrays) as other investigators have done [64]. Our stringent quality control approach and the genetic matching minimized the number of false positive associations, likely at the expense of missing some true positive associations. We decided to use genetic matching to reduce the effect of population stratification because our initial genome-wide association study that included all control subjects from the Illumina repository had a genomic control factor >1.3 suggesting substantial population stratification between cases and controls. Simulation studies that we published in [65] showed that matching is a good way to remove the effect of stratification without losing too much power. In addition, a traditional model that includes principal components from genome-wide principal component analysis would not be useful for prediction because the values of the principal components for new subjects would be missing. Our analysis does not show any systematic difference between results in the controls genotyped in our lab compared to healthy controls genotyped elsewhere (**Figures 5** **and** **8**). Also, additional analyses using traditional principal-components approaches to control for population stratification suggest that no residual stratification is likely to confound the associations (**Figures S9 and S10**). However, only replication of these results in independent data from comparably old subjects by independent investigators will definitively validate the results and this approach.

In our study we included only Caucasian subjects and the extent to which this analysis applies to other racial groups is an open question.

### Novel insights about the genetics of exceptional longevity

The large number of SNPs in our genetic risk model and the variety of genetic signatures confirm that exceptional longevity is influenced by the combined effects of a large number of SNPs. The genetic risk model implicates 130 genes, most of them known to play a role in various disease mechanisms (**Figure 5**), and our findings suggest that different variants of these genes may have a protective role. The most intriguing examples are *LMNA* and *WRN*: while specific variants of these two genes determine progeria and accelerated aging, alternative variants may increase life span. About 50% of the SNPs in the genetic risk model are in intragenic regions and this also suggests that regulatory mechanisms play an important role in exceptional longevity. We also found that the sensitivity of the prediction in independent sets increases with the ages of centenarians, and therefore likely, the genetic contribution to lifespan increases with increasing ages of the centenarians.

Our analysis provides further insight about the role of *APOE* in survival to extreme ages. Although the SNP rs2075650 in *TOMM40/APOE* is the most significantly association with exceptional longevity, the value of this SNP to identify who can live to 100 and older appears to be limited. The traces of sensitivity and specificity of the nested genetic models in **Figure 4B** show that the model with only this SNP has 85% sensitivity to predict exceptional longevity but only 26% specificity in the discovery set. We conducted an ROC analysis to show the poor predictive value of this SNP alone (**Figure S19**, AUC = 0.62). Also, sensitivity and specificity of the model with only this SNP are 85%/26% in the ELIXIR set, and 82%/23% in the second replication set. The traces of sensitivity/specificity of the models with increasing number of SNPs show that, the predictive accuracy increases only when a substantial number of variants are added to the model that includes rs2075650 (**Figure 4B**). We also examined the changes in sensitivity/specificity when we removed this SNP from the list of 281, and dropping rs2075650 resulted in a loss of approximately 1% accuracy (88% sensitivity/specificity in the discovery set (AUC = 0.95); 55% sensitivity and 58% specificity in the ELIX set (AUC = 0.56); and 75% sensitivity and 60% specificity in the additional 60 centenarians and 2863 Illumina controls (AUC = 0.73)) These results are summarized in **Figure S7**. This SNP is only in weak linkage disequilibrium with the two SNPs that define the 3 alleles of *APOE* but its association with longevity was shown to be dependent on the APOE alleles in [66]. The reason for the low predictive value of rs2075650 alone is that the GG genotype of this SNP is rare in the population (genotype frequency 3%) but virtually absent in centenarians (genotype frequency 0.1%), therefore if someone is a carrier of the GG allele it is unlikely that he will become a centenarian, while predicting the outcome in carriers of the AA or AG genotypes is more difficult without additional genetic data.

The NECS previously showed that centenarians fall into different groups in terms of age of onset of age-related diseases: survivors (onset of aging disease ≤80 years), delayers (onset of aging disease between 80 and 100 years) and escapers (age of onset ≥100 years) [67]. This current analysis now shows that some of the centenarians carry genetic signatures that correlate with different ages of survival and suggests that the complexity of aging and the different patterns of survival to the age of 100 and older may be the result of different genetic profiles. Unlike the typical approach of finding individuals with a specific phenotype in common and then performing a genetic association study to discover genetic associations with the trait, our approach tries to dissect a complex phenotype into sub-phenotypes based on the genetic data. Our analysis is preliminary, based on small a sample, and needs to be replicated but we hope that this new approach may prove useful in dissecting other complex genetic traits [68].

**Figure 10:**
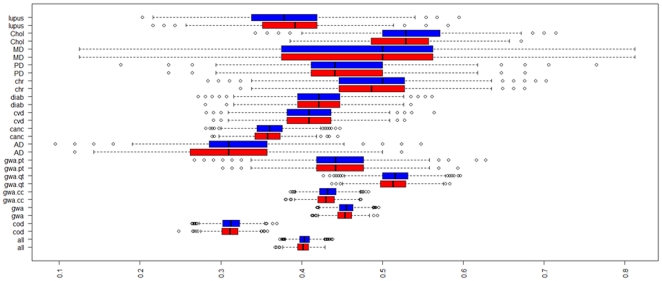
Distribution of risk alleles of 1214 SNPs in 1054 centenarians (red) and 4118 controls (blue). Risk alleles were derived from the GWAS catalogue at the NHGRI (downloaded in April 2011) and the Human Genome Mutation Database. The boxplots displays the rate of risk alleles carried by centenarians (red) and controls (blue). The disease described are: lupus, cholesterol level (Chol), macular degeneration (MD), Parkinson's Disease (PD), Chron's disease (chr), diabetes (diab), cardiovascular disease (CVD), cance (canc)r, Alzheimer's (AD), GWAS.pt is the group of alleles related to personality disorders that were found in GWAS, gwas.qt is the group of alleles related to QTL from GWASs and include cholesterol, BMI, obesity etc, and GWAS.cc is the group of risk alleles found from case/control GWASs so include for example cancer, PD, MD etc, cod is for coding variants from the HGMD, and all is the full set of 1214 variants. Table S3 reports the actual rates.

While large numbers of longevity associated variants appear to be necessary for extreme survival, we did not observe a substantial difference in the numbers of a large sample of known disease-associated variants carried by centenarians and controls (**Figure 10** **, Table S3**). The Leiden Longevity and Leiden 85+ Studies recently produced similar findings for alleles associated with specific age-related diseases amongst 85+ year olds and nonagenarians [69]. Furthermore, only 13 SNPs previously associated with common diseases in genome wide association studies reach statistical significance in the discovery set, and the risk alleles are significantly less frequent in centenarians than in controls (**Table S4**) [70], [71], [72], [73], [74], [75].

These preliminary data suggest that exceptional longevity may be the result of an enrichment of longevity associated variants that counter the effect of disease-risk alleles and contribute to the compression of morbidity and/or disability towards the end of very long lives [43].

In our analysis we also found that specific signatures correlated with the prevalence and age of onset of some age-related diseases and further investigation is needed to understand how and why they predispose for exceptional longevity and for specific, different patterns of aging. The genetic signatures were built by using an ensemble of genetic risk models. The high sensitivity of these predictions in independent samples of centenarians shows that genetic data can indeed predict exceptional longevity without knowledge of any other risk factors. The high sensitivity is consistent with (1) theoretical results that show potentially high predictability of rare and highly heritable traits even when only 50% of the genetic variants that determine the trait are found [36] and (2) the accuracy of genetic risk models that have been developed to predict complex and highly heritable traits [37], [38], [39], [40], [41]. To quantify the amount of genetic variance in liability to exceptional longevity that is explained by our model, we used the online calculator http://gump.qimr.edu.au/genroc/ to translate the predictive accuracy measured by the AUC in proportion of explained genetic variance on the liability scale [36]. Based on previous reports and the latest US 2010 Census (http://www.census.gov/prod/cen2010/briefs/c2010br-03.pdf), we estimated that the prevalence of exceptional longevity (living to 100+) is 1 in every 5,000 people, while the sibling relative risk for exceptional longevity ranges between 8 and 17 [9], [10]. With these numbers, we estimated that the maximum AUC of a genetic model of exceptional longevity ranges between 0.95 to 0.98 and our genetic model that reaches AUC = 0.74 in the second replication set (**Figure S7**) explains between 12% to 17% of the genetic variance on the liability scale. In the ELIXIR replication set, the AUC of our genetic risk model is 0.58 and this would represent 1–2% of explained genetic variance. Since the ELIXIR set includes more nonagenarians than centenarians, and their prevalence in the population is 0.5% and the sibling relative risk of this trait is approximately 2.5, we repeated the calculations in this scenario and the 0.58 AUC translated into approximately 4% of the genetic variance in the liability scale. These results show that although we explained a good amount of genetic variability on the liability scale to live to very old ages, there is still more than 80% missing heritability that remained to be explained, and more comprehensive genetic studies have the real potential to decipher the genetic base of this complex phenotype.

Some centenarians in our study however lack a genetic signature conducive to exceptional longevity. The strong clustering of exceptional longevity in some of their families suggests that these individuals harbor rare or private alleles associated with exceptional longevity. This in turn would suggest that sequencing these individuals could be particularly fruitful.

The specificity of our classification rule is 60–61% in the independent sets and is comparable to other genetic studies of complex traits [76], [77], [78]. Although the specificity is better than random, it would not be useful as a diagnostic test. The decreased specificity in this study could be explained by the fact that the control subjects from the Illumina database are primarily made up of healthy controls used for other genome-wide association studies and therefore the control data set may be enriched for healthy aging subjects.

Our finding that about 17% of Illumina controls have signatures with >70% chance of exceptional longevity (**Figure S14**) suggests that a substantial proportion of this group have a genetic predisposition to exceptional longevity. If this observation is replicated in more representative samples of the population, it could in part explain why centenarians are the fastest growing age group in developed countries [79], [80]. At the turn of the last century, infant mortality was approximately 25%. As public health measures markedly reduced infant mortality rates in the first quarter of the 20^th^ century, a greater and greater proportion of the population had the opportunity to age into middle and older ages. If nearly one fifth of the population had an increased genetic predisposition to survive to 100 years, it is understandable why the number of centenarians is growing at such a relatively high rate.

Although sensitivity and specificity of our classification rule may improve with a more comprehensive knowledge of human genomic variation, its limitations could also suggest that environmental factors (e.g., lifestyle) contribute in important ways to the ability of people to survive to very old ages. Replications of these results in independent cohorts will help to answer these questions.

## Materials and Methods

### Ethics statement

NECS and Elixir subjects were enrolled under similar protocols approved by Boston Medical Center's Institutional Review Board and the Western Institutional Review Board, respectively. Written informed consent was obtained for all NECS and ELIXIR subjects.

### Study populations

The New England Centenarian Study (NECS) began in 1994 as a population-based study of all centenarians living within 8 towns in the Boston area [81]. Since ∼2000, the NECS expanded enrollment to include centenarians from throughout the USA (www.bumc.bu.edu/centenarian). Potential subjects are ascertained via voting records and media alerts. Subjects are sent a demographic data, life style choices, medical history and functional status questionnaire, family pedigree form and blood kit. A dementia scale test is administered over the telephone. The study is still actively recruiting centenarians, with an average of 50 subjects enrolled per year.

#### Elixir Pharmaceuticals American Centenarians

In 2001–2003, Elixir Pharmaceuticals (co-founded by Leonard Guarante and Cynthia Kenyon) conducted a U.S. nation-wide centenarian recruitment effort. Since 2006, Elixir's centenarian research effort has ceased (and DNA and data are stored and have also been shared with the NECS, where genotyping of all the samples was performed in 2008). Recruitment and data collection were modeled after the NECS protocol.

#### NECS controls

The NECS has recruited approximately 450 referent subjects comprised of spouses of centenarian offspring and children of parents who died at the mean age of 73 years, with an age at enrollment ranging between 53 and 90 years.

#### Illumina controls

We identified 3,613 Caucasian healthy controls from the Illumina control database (iControlDB, http://www.illumina.com/downloads/PurposeDocument.pdf). No phenotypic information is available for subjects selected from the Illumina repository, except for gender (∼60% females) and age at blood draw for some subjects (age range 0—75 years).

The Coriell NINDS control sample in the Parkinson's disease (PD) set is described elsewhere [21].

Subjects from these studies were combined to generate a discovery and replication set using genetic matching (see below) and an additional replication set in which subjects were not genetically matched.

#### Discovery set (NECS)

This consisted of 801 cases and 914 controls. Cases are long lived individuals from the NECS who were born between 1880 and 1910 and reached an age at death between 95 and 119 (mean 104±3, median 104). Controls were comprised of 673 healthy controls from the Illumina database (Illumina I), and 241 referent subjects from the NECS. Controls were selected to match the genetic background of cases.

#### Replication 1 (ELIX)

This is comprised of 253 long lived individuals enrolled from ELIXIR Pharmaceutical (mean age 101±3, median 100), and 341 healthy controls from the Illumina database (Illumina II). Controls were selected to match the genetic background of the 253 cases in this set.

#### Replication 2 (NECS 2)

60 NECS individuals and 2863 healthy controls from the Illumina database (Ilumina III). In this set, no genetic matching was performed. The 60 centenarians include 39 subjects of European ancestry enrolled between June 2009 and September 2010 (age range 100–114, mean age 108) plus 21 centenarians also of European ancestry (age range 101–115, mean age 107) that were not included in the discovery set during the genetic matching.

### SNP genotyping

We analyzed 1 ug of genomic DNA for NECS and ELIXIR samples, using the Illumina 370 CNV chip, v.1, the Human610-Quad v1.0, and the Human 1 M v1.0 (Illumina, San Diego, CA). We used the Beadstudio software for genotype calling using the top-strand rule, so that SNPs alleles are coded using lexicographical order (typically A/G and A/C). The data in the Illumina repository were generated with different SNP arrays (300 and 550) and we selected the SNPs that were in common to all platforms. SNPs with reverse alleles, and monomorphic in some of the arrays were detected by comparing allele frequencies in controls (300 vs 550, 370 vs 550), and in centenarians (370 vs 1 M, 370 vs 610). **Table 2** summarizes the arrays used.

**Table 2:** Breakdown of genotyped samples by Illumina SNP array type (columns 3—7), laboratory (column 8), and case/control/study status (rows).

		370	610	1 M	300	550	Lab
**Centenarians**	**NECS**	583	102	176	0	0	BU
	**ELIXIR**	209	44	0	0	0	BU
**Controls**	**NECS**	237	4	0	0	0	BU
	**Illumina I**	0	0	0	89	584	unknown
	**Illumina II**	0	0	0	62	279	unknown
	**Illumina III**	0	0	0	574	2289	unknown
	**Coriell NINDs**	867	0	0	0	0	CIDR

The columns of the table denote the Illumina array types. The column “Lab” denotes the laboratory that performed the genotyping: BU = Boston University; CIDR = Center for Inherited Disease Research. The row Illumina I denotes the control samples included in the discovery set; Illumina II denotes the control samples included in the first replication set, and Illumina III denotes the residual samples from the Illumina repository; Coriell NINDs denotes the neurologically normal controls.

### Quality Control

#### Rules for sample inclusion

Raw GWAS data were clustered using standard Illumina cluster definitions in array-specific batches (all 370 samples together, all 1 M samples together, all 610 samples together). Specifically, we performed sample-based QC checks and produced QC statistics to compute sample call rates (CR). We eliminated all samples with CR<96.5% and remaining samples were reclustered. After re-clustering, we included the “excluded” samples using this new cluster file. If the previously excluded samples had a CR above 93% they were included in the final analysis.

We also used the genome-wide identity by descent analysis in PLINK [82], to discover unknown relatedness and to estimate error rate using the number of mismatch of replicated samples (2%). With this analysis we discovered one subject enrolled in both the NECS and ELIX studies, whom we removed from the ELIX set. We also removed samples with inconsistent gender between heterozygosity of the X chromosome and gender recorded in the database.

#### Rules for SNP inclusion

SNPs were included in the final clean data set if all these conditions were satisfied:

1. CR>98% in each array type (300, 370, 550, 610, 1 M) in both centenarians and controls of the discovery set, and overall CR>98% in all samples included in discovery and replication sets.
2. Cluster separation score >0.25.
3. Excess heterozygosity score between −0.3 and 0.3.
4. Hardy Weinberg equilibrium χ^2^ statistics in controls <50.
5. Minor allele frequency difference between any pair of array type <0.2

A total of 243,980 SNPs were selected for the analysis.

#### Assessment of between arrays bias and batch effects

The 610-Quad is part of the new line of Infinium high density whole-genome genotyping products, and had undergone substantial design changes compared to the Human CNV370, Human 1 M, HumanHap550-Duo and HumanHap300. We used data from 32 samples that had been genotyped with both the Human CNV370 and 610-Quad illumina arrays and that underwent the same QC procedure, to test for systematic bias between the two arrays. 345,219 SNPs were in common between the two arrays but only 294,153 SNPs had CR>0.97 (so at least 31 genotypes were called) in both arrays after reclustering. In this set, 915 SNPs had 2 or more different genotypes, and only 28 SNPs had allele frequencies that differed by more than 0.05. The plot of allele frequencies (**Figure S20**) suggests that there is no systematic bias between arrays but rather sporadic errors that can be identified by plotting allele frequencies.

We tested the agreement between allele coding in the other arrays by comparing the allele frequencies. See **Figure S21**. The plots rule out general bias between arrays and show that SNPs with reversed alleles were removed.

The additional sample of 60 centenarians included 39 subjects that were genotyped in September 2010, using the 610-Quad array. To be able to test for batch effects, we genotyped the 39 samples in a batch of 48 that included two replicated samples, and 7 samples that had been genotyped with the Human 1 M in the original analysis. The agreement between genotype calls in the 7 samples genotyped with the 610-Quad and the Human 1 M ranged between 99.2% and 99.7%.

### Genetic matching of controls

Population stratification was assumed to be a serious problem with the centenarian and control data, because a large proportion of NECS subjects were immigrants from Europe, and the patterns of immigration at the end of the 19^th^ century may lead to an overrepresentation of some European ethnic groups [83]. In fact, an initial GWAS analysis in which we randomly selected controls from the Illumina repository pointed to substantial stratification (genomic control factor ∼1.3). We therefore reduced possible confounding due to population stratification by selecting controls to match the genetic backgrounds of NECS subjects.

To identify the population substructure in the centenarians and controls we ran a principal components analysis with the software EIGENSOFT [84], using GWAS SNP data for SNPs common to the NECS and Illumina datasets that had a SNP call rate>0.95 and MAF>0.05. SNPs in strong LD were removed using the program PLINK with a SNP window of 50 and sliding window of 5 SNPs and we removed 1 SNP from each pair of SNPs with r^2^>0.30 leaving 97,508 SNPs for this analysis. We found that the top several principal components (PCs) correlated to the genetic ancestry and formed a similar pattern to other studies of subjects of European ancestry [84], [85]. However, the analysis also showed that the Illumina controls contain many more ethnic groups than the NECS (**Figure S1**), and the inclusion of these control subjects might therefore inflate false positive associations. We used the clustering algorithm in [65] to group individuals with similar ancestry into the same cluster. The algorithm utilizes k-means clustering to iteratively group individuals into cluster sizes varying from 2 to 30 and then computes a scoring index at each cluster size that accounts for the accuracy of the subjects' cluster assignments, the stability of k-means clustering from iteration to iteration and the ability of the algorithm to maximize the distance between subjects allocated to different clusters. This analysis identified 20 clusters corresponding to sub-populations with different genetic structure, and **Figure S1** shows the details of the clusters and their ethnic labels based on the known mother tongue and ancestry of the cluster members. NECS cases were present in only 16 of the 20 clusters as shown in **Table 3** that displays the frequency of NECS cases (row 2), NECS controls (row 3) and Illumina controls (row 4). For example, no centenarians were allocated to cluster 1 or 15 (empty and full red dots in **Figure S1** that may represent Franks and Celtics- Alpine ethnicities). To increase the number of controls, we randomly selected additional Illumina controls from those 16 clusters to maintain the same ratio of cases/controls in each cluster. For example, we sampled 4 additional Illumina controls from cluster 2, so that the ratio case/controls in cluster 2 was 21/24 = 0.88, and similarly, we sampled 19 additional controls from cluster 9, so that the ratio case/control in cluster 9 was 31/35 = 0.88 etc.

**Table 3:** Distribution of NECS cases (row 2), NECS controls (row 3) and Illumina controls (row 4) in clusters of genetic ethnicity (columns).

	1	2	3	4	5	6	7	8	9	10	11	12	13	14	15	16	17	18	19	20
Cent	0	21	34	79	27	189	6	0	31	102	22	20	3	94	0	15	94	34	0	25
Control	2	20	8	14	30	38	2	1	16	19	18	3	4	12	4	3	29	7	0	12
Illumina	90	310	192	47	278	168	223	104	277	288	200	120	173	132	169	54	266	154	118	250

The table shows the 20 clusters of genetic ethnicity that were discovered using a clustering algorithm described in reference [20]. Note that no centenarians were allocated to cluster 1 or 15. These clusters are represented by full red dots in **Figure S1** and denote Franks and Celtics- Alpine ethnicities.

### Single SNP Analysis

#### Bayesian test of association

We employed both Bayesian and traditional frequentist analyses of four different genetic models: general in which we analyzed the distribution of three genotypes; allelic in which we analyzed the distribution of alleles M versus m; recessive and dominant in which we grouped the genotypes in two groups, either MM/Mm versus mm (dominant for TOP strand allele), or MM versus Mm/mm (recessive for TOP strand allele) respectively. Note that M is the allele in the TOP strand and m is the allele in the BOTTOM strand based on Illumina genotype calling rules. We used a traditional χ^2^ test of independence in a 2×3 contingency table to test general association, and the χ^2^ test of independence in a 2×2 contingency table to test additive, dominant and recessive associations.

With the Bayesian analysis, we scored each SNP association by the Bayes Factor (BF) that can be interpreted as the posterior odds for the association when the null hypothesis of no association and the alternative hypothesis of an association have the same prior probability [86]. Specifically, let H0 and H1 denote the null hypothesis of no association between the SNP and the phenotype and the alternative hypothesis that there is an association between the SNP and the phenotype, and let p(H0) and p(H1) denote the prior probabilities of the two hypotheses. Then, by Bayes' theorem, the posterior odds of the alternative hypothesis is computed as:
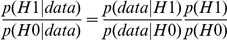
The quantities p(data|H0) and p(data|H1) are the “marginal likelihoods” of the data, given the two hypotheses H0 and H1, and are computed as the solutions to the two integrals
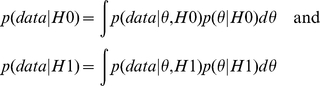
The quantities 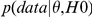 and 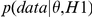 are the traditional likelihood functions under the null and alternative hypotheses, and 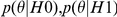 are the prior distributions of the parameters of the two likelihood functions. These parameters are the conditional probabilities of the SNPs alleles in cases and controls and, in the paragraph below, we will provide details of the parameterizations. The ratio p(data|H1)/p(data|H0) is the BF, so that under the assumption that p(H0) = p(H1) = 0.5, the posterior odds equals the BF. The BF can be computed in closed form for all 4 models when appropriate parameterizations are used and missing genotypes are assumed to be missing at random [87]. The formulas are given below.

We assume that genotypes frequencies in cases and controls follow independent multinomial distributions with parameters that follow Dirichlet distributions with uniform prior hyper-parameters. This is the standard parameterization for conjugate Bayesian analysis of a contingency table when we condition on one dimension of the table. See for example the supplement material of the review article of Balding [86]. In our case, we condition on the phenotype (case/control status) so that we use the retrospective likelihood that is appropriate in a case-control design.

Then the marginal likelihood of the data, given a genotype association, is the formula:
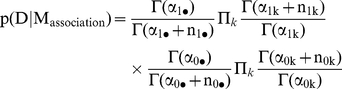
and the marginal likelihood of the data, assuming no association between SNP and phenotype, is the formula:
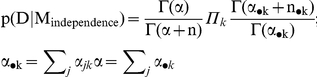
where the genotype frequencies 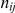 and hyper-parameters 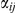 of the Dirichlet distribution are defined in **Table 4** and **5**. The Bayes factor is the ratio between the two marginal likelihoods:
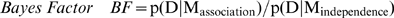
The Bayes factors for the other models are calculated using the same formulas, after the genotype frequencies are converted into allele frequencies (**Table 6**), or frequencies for dominant alleles (**Table 7**), and recessive alleles (**Table 8**).

**Table 4:** Notation of genotype frequencies.

	Genotype Frequencies			
	MM	Mm	mm	Total
Cases (Y = 1)	n_11_	n_12_	n_13_	n_1_.
Controls(Y = 0)	n_01_	n_02_	n_03_	n_0_.
Total	n._1_	n._2_	n._3_	N

The table defines the mathematical notation for the genotype frequencies used in the methods.

**Table 5:** Notation of the hyper-parameters in the Dirichlet prior distributions.

	Prior Hyper-parameters			
	MM	Mm	mm	Total
Cases (Y = 1)	α_11_	α_12_	α_13_	α_1_.
Controls(Y = 0)	α_01_	α_02_	α_03_	α_0_.
Total	α._1_	α._2_	α._3_	α

The table defines the mathematical notation for the hyper-parameters of the Dirichlet distribution used in the methods.

**Table 6:** Notation of allele frequencies in the allelic model.

	Allele Frequencies		
	M	M	Total
Cases (Y = 1)	2n_11_+n_12_	n_12_+2n_13_	2n_1_.
Controls(Y = 0)	2n_01_+n_02_	n_02_+2n_03_	2n_0_.
Total	2n._1_+n._2_	n._2_+2n._3_	2N

The table defines the mathematical notation for the allele frequencies used in the methods.

**Table 7:** Notation of allele frequencies in the dominant model.

	Allele Frequencies		
	MM/Mm	Mm	Total
Cases (Y = 1)	n_11_+n_12_	n_13_	n_1_.
Controls(Y = 0)	n_01_+n_02_	n_03_	n_0_.
Total	n._1_+n._2_	n._3_	N

The table defines the mathematical notation for the dominant model for the M allele in the methods.

**Table 8:** Notation of allele frequencies in the recessive model.

	Allele Frequencies		
	M	M	Total
Cases (Y = 1)	n_11_	n_12_+n_13_	n_1_.
Controls(Y = 0)	n_01_	n_02_+n_03_	n_0_.
Total	n._1_	n._2_+n._3_	N

The table defines the mathematical notation for the recessive model for the M allele in the methods.

We used α_jk_ = 2 in all 4 tests.

For genotype association, we estimated the two ORs for exceptional longevity (EL) as:
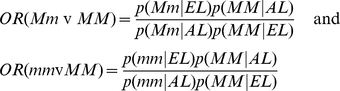
And we estimated the conditional probabilities of genotypes as:
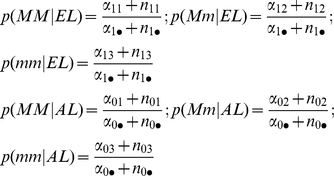
The formulas are similar for the other genetic models. We estimated the genomic control factor as described in [88].

#### Interpretation of MBF

We conducted extensive simulations to compute the expected number of false positive associations of the decision rule that selects a significant association when the BF of at least one of the four models is greater than the threshold. For each allele frequency p(a) = 0.05,0.10,0.15,…,0.5, we simulated 100,000 data sets with no associations with 1750 subjects that we randomly split into 800 cases and 950 controls, to mimic the sample size of the discovery set. We used thresholds varying between 10 and 1,800 and, in each simulated data set, we computed the BF for the 4 models of association as described above, and determined the SNP as significantly associated if the BF of at least one of the 4 models was greater than the threshold. The simulations are summarized in **Figure S2** and show how to interpret different thresholds for the MBF in terms of expected error rate.

#### Gender effect

For the significant SNPs in the discovery set, we tested whether the associations are substantially modified when a gender-SNP interaction model was used. We used the retrospective likelihood and tested whether the distribution of each selected SNP is independent of the phenotype once we condition on gender. Accepting the null hypothesis implies that the association between SNPs and phenotype is explained away by gender and none of the associations could be explained away by gender.

#### Association with known disease alleles

We identified 62,339 unique SNPs that were associated with a variety of diseases and traits in several GWASs from the catalogue of published genome wide association studies at http://www.genome.gov/26525384 [89], and the Human Gene Mutation Database (HGMD). We found 1214 of these SNPs in the Illumina array that we used for the GWAS of EL with acceptable quality. We calculated the number of disease alleles carried by centenarians versus all Caucasian controls included in our analysis.

### Genetic Risk Modeling

#### Nested Bayesian Models

We define k nested SNP sets (k = 1,…,K), starting from the most significant SNP
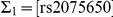
and then we increment the set by adding one SNP at a time in order of maximum Bayesian factor (MBF). The latter is the maximum Bayes Factor among the 4 genetic models that we tested for each SNP in the GWAS. Therefore, the (k+1)th SNP set is defined as
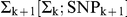
where SNP_k+1_ is the SNP with the (k+1)th Bayesian significance. We choose K = 500 that corresponds to testing SNPs with approximately a posterior probability of an association >0.95, and removed from this set 100 SNPs that are highly correlated. To this end, we build a Bayesian network to capture mutual dependencies between SNPs that represent either strong linkage disequilibrium or strong SNP-SNP associations and removed those SNPs that are conditionally independent of the phenotype given more significant SNPs. We used a threshold on the posterior probability of association ranging between 10 for multiple dependencies to 100 for two-way SNPxSNP interaction. The methodology based on Bayesian networks is described in details in [62], [90].

For each SNP set, Σ_k_, the Bayesian classification rule calculates the posterior probability of EL as:
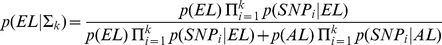
where p(EL) and p(AL) = 1-p(EL) are the prior probabilities of exceptional and average longevity. The conditional probabilities 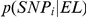 and 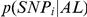 represent the distribution of the ith SNP genotype in cases (EL) and controls (AL). The rule is to classify a subject as predisposed to exceptional longevity if 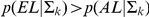.

We used the prior Pr(EL) = Pr(AL) = 0.5 as described in the caption of Figure 4. This choice of a prior probability 0.5 for both EL and AL means that the classification becomes independent of the prior because, by Bayes' theorem, the rule becomes “assign EL” if
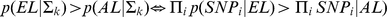
and hence when the probability of the data given EL is greater than the probability of the data given AL.

The quantities
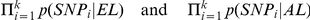
are the joint probabilities of the SNPs in the set Σ_k_ that are estimated from the cases and controls. The rationale of this formula is that the SNPs are modeled as conditionally independent given the phenotype so that the probability distribution of a SNP set, given the phenotype, has the product form
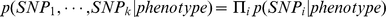
The product form is equivalent to assuming that the SNPs have a multiplicative effect, as in an additive logistic regression model. Compared to logistic regression, the Bayesian classification rule uses the retrospective likelihood to update the prior probabilities of EL and AL into the posterior probabilities. Also, the product form in the retrospective likelihood has the advantage that the genetic effect of each SNP can be estimated independently of the other SNPs and so there is virtually no upper limit on the number of SNPs that we can include in the SNP set.

We estimate the conditional probabilities 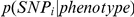 using conjugate Bayesian analysis as described earlier.

#### Evaluation of sensitivity and specificity

Sensitivity (how many centenarians are predicted as centenarians) and specificity (how many controls are predicted as controls) of each SNP set were estimated as:

Sensitivity = proportion of centenarians in the discovery set for whom p(EL | Σ_k_)≥p(AL | Σ_k_);
Specificity = proportion of controls in the discovery set for whom p(EL | Σ_k_)<p(AL | Σ_k_);

#### Resampling Method

In the bootstrap-type approach, we repeatedly split the discovery set into non overlapping 2/3 training and 1/3 test sets that were respectively used to estimate the nested genetic risk models and to evaluate their predictive value. We repeated this random procedure 1000 times for each SNP set, and summarize the sensitivity and specificity into the average values (See **Figure S4**). We evaluated the growth of sensitivity and specificity in the 1000 resampled sets. The mean number of SNPs in which the absolute difference between sensitivity and specificity was <0.02 and accuracy was >85% was 281.

#### Effect of the search order

We tested the effect of our ordering heuristics to see whether different orderings may lead to better risk prediction models. We conducted two types of tests. In the first test, we randomly permuted the order of the top 281 SNPs and repeated the heuristic of building nested genetic risk models by adding one SNP at a time from the randomized list of SNPs. In each test, we examined the effect of changing SNP order on the sensitivity and specificity in the discovery set, and also in the bootstrap procedure. The results of these analyses are shown in **Figure S4**.

#### Interpretation of genetic risk profiles

We generated genetic risk profiles for each subject by plotting the posterior probability of EL (p(EL|Σ_k_), y axis) against the number of SNPs in each of 281 SNP sets (x-axis). The trend of the profiles informs about the enrichment of longevity associated variants (LAV)s because the posterior probability of exceptional longevity in a subject, given the SNP set Σ_k+1_ is greater than that given the SNP set Σ_k_ if the subjects carries a genotype of the (k+1)th SNP that is more common in centenarians rather than controls. In fact
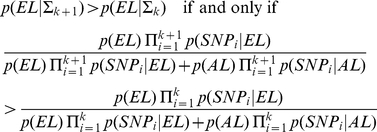
And the inequality is equivalent to
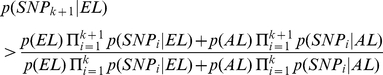
This can be written as
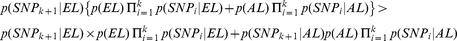
which simplifies into
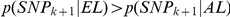
Note that this property is independent of the SNPs in the current SNP set, so changing the order of the nested model may change the overall pattern of the risk profile but not the interpretation in terms of enrichment of longevity associated variants.

#### Ensemble of genetic risk models

The ensemble of genetic risk models uses 281 nested SNP sets to compute the risk for EL and AL (average longevity), and the overall risk is estimated as the average of all genetic risks:



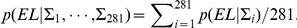



#### Prediction in independent tests

For prediction, we used the ensemble of 281 genetic risk models trained in the discovery set and computed the posterior probability of AL and EL in cases and controls of the two sets. We assumed uniform prior probabilities (P(AL) = P(EL) = 0.5), and classify a subjects as EL if the posterior probability of EL given the genotype of 281 SNPs was>posterior probability of AL. We assessed sensitivity and specificity by the number of centenarians classified as EL and the number of controls classified as AL.

### Genetic Signatures

#### Clustering of genetic risk profiles

We used the Bayesian model-based clustering procedure implemented in the program CAGED [91] to cluster the genetic risk profiles of centenarians and controls, independently, in the discovery and replication sets. The method in CAGED is designed to cluster row profiles of a two dimensional array by preserving the column ordering and it uses a Bayesian search strategy to identify the number of clusters by maximizing a Bayesian score [92]. We organized the genetic risk profiles into a N x 281 array, with rows that represent subjects and the jth column that represents the genetic risk calculated from the jth SNP set. We used polynomial models up to order 4 to capture a variety of patterns [93] and then used hierarchical clustering of the profiles to check whether similar clusters could be further merged. The signatures in the merged replication sets were generated by cluster analysis of the predicted profiles calculated using the 281 genetic risk models trained in the discovery set.

#### Correlation of genetic signatures with aging subphenotypes

Difference in survival was tested using log-rank tests implemented in the survival package of R. Only subjects with events or alive without events were included in the analysis.

#### Correlation of genetic signatures with race and ethnicity

We tested the association between the genetic signatures in centenarians and the genetic structure determined with the cluster algorithm of the principal components. When we correlate the 26 clusters of genetic signatures to the clusters of different population structures, we did not find any association (the p-value from χ^2^ was 0.3).

#### Validation with the TaqMan platform

In order to validate the genotyping of the SNPs included in the model, 30 SNPs (>10% of the SNPs in the model) were selected to re-genotype using the TaqMan platform (Applied Biosystems, Carlsbad, CA). This genotyping was performed at Yale University. The samples included 688 centenarians and 221 controls from the NECS that were included in the discovery set and for whom we had available DNA. For each sample, 2.5 ng of DNA was arrayed into 384-well plates and was dried prior to TaqMan genotyping. Thermal cycling was performed using either a BioRad C1000 or S1000 (BioRad, Hercules, CA) and plate reads were done using the CFX Optical Reaction Module (BioRad, Hercules, CA). Genotype calls were made using the BioRad CFX Manager Software for Allelic Discrimination (BioRad, Hercules, CA). Of the 30 SNPs attempted, 28 SNPs were successfully genotyped; one zSNP, rs4802234, did not yield data that could be clustered using the allelic discrimination software for one of the three 384-well plates, and one SNP, rs12629971, had a lower call rate (93%). All TaqMan genotyping was performed blind to the microarray genotypes as the Yale group did not have access to the microarray genotypes.

There were 34 duplicate samples genotyped using TaqMan across the 28 SNPs generating a total of 952 duplicate genotypes, 950 of which had both samples called. Of these 950 duplicate genotypes, 100% of the genotypes were concordant. For the 28 SNPs successfully genotyped, we observed between 1 and 10 discordant genotypes per SNP between the TaqMan genotype and the microarray genotype, yielding concordance rates between 98.88 and 99.89% between genotyping platforms. Our overall discordance rate across all SNPs was <1%.

This low rate of discordant genotypes did not affect the results: 23 of the 28 SNPs reached statistical significance in the replicated data, and although 5 SNPs did not reach statistical significance possibly because of the small sample of controls, the allele frequencies from the microarray data and TaqMan data are virtually indistinguishable (**Figure S5**), suggesting that a 1% genotyping error rate should have no impact on this analysis.

## Supporting Information

Figure S1
**Population structure of centenarians and controls.** Scatter plot of principal components 1 and 2 (PC1 and 2 PC2, top panels), and principal components 3 and 4 (PC3 and PC4, bottom panels) in subjects from the NECS (left) and Illumina database (right) that were estimated using genome wide data. We labeled the clusters by ethnicity using the information about mother tongue and place of birth of NECS subjects and their parents. Note that some of the European ethnic groups in controls (NECS and Illumina) are not represented in NECS cases, for example Italics (⊗ green), Saxon/Scandinavia (• green), Celtics/Alpine (▪ red), and Franks (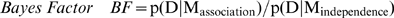, red).(TIF)

Figure S2 **Error rate in log10 scale of the Bayes rule for different thresholds of the MBF.** The x axes reports the estimate of the −log10(error rate) and 95% credible intervals that were estimated using a Beta distribution in 1,000,000 simulations per threshold on the MBF (y-axis). The MBF is the maximum Bayes Factor computed to test the association of each SNP in 4 genetic models (genotypic, allelic, dominant, recessive). The genotype data were generated with allele frequencies varying uniformly between 0.05 and 0.5 and assuming HWE. The analysis suggests that a MBF>1,400 determines an error rate of approximately 1 to 2 errors per 100,000 tested association (−log10(2/100,000) = 4.7)), and a MBF>100 determines an error rate of approximately 4 errors per 100,000 tested association (−log10(4/100,000) = 3.4). Note that this analysis includes the additional costs of searching for 4 genetic models.
(TIF)

Figure S3 **Manhattan plot and QQ-plot for the allelic association tested using a traditional frequentist approach.** The Manhattan plot shows the −log10(p-value) for the 1 degree of freedom test Chi-square test. The QQ-plot displays the observed quantiles of the 1 degree of freedom test Chi-square test versus the expected quantiles.
(TIF)

Figure S4 **Effect of sampling variability, and SNP ordering on the sensitivity and specificity of the model.** **Panel A)** displays the average sensitivity and specificity of 400 nested models in 1000 resampled sets. 1000 training and test sets were randomly resampled from the discovery set and each training set was used to estimate the Bayesian classification rule that was tested in the test set. The plot displays the average sensitivity and specificity (y-axis) versus number of SNPs (x-axis). The sensitivity is the proportion of centenarians with posterior probability of exceptional longevity>posterior probability of average longevity and the specificity is the proportion of controls with posterior probability of exceptional longevity<posterior probability of average longevity. The mean number of SNPs in which the absolute difference between sensitivity and specificity was <0.02 and accuracy was >85% was 281. **Panel B)** displays the specificity for the two types of controls in the discovery set (NECS referent subjects: continuous line; Illumina controls: dashed lines) and shows that there is no difference between the two control sets. **Panel C)** describes the effect of re-ordering the 281 SNPs. Patterns of sensitivity and specificity using the discovery set (left), and randomly generated validation sets (right) when the top 281 SNPs were randomly entered into the nested models (continuous lines: SNPs are ordered by MBF; dashed lines: the same 281 SNPs are randomly arranged). **Panel D)** describes the effect of random selection on sensitivity and specificity of the nested models. Patterns of sensitivity and specificity using the discovery set (left), and randomly generated validation sets (right) when 281 SNPs were randomly chosen from the top 1,700 most significant SNPs. (continuous lines: SNPs are ordered by MBF; dashed lines: 281 SNPs are randomly selected from the 1700 most significant). The analysis shows that changing the order affects sensitivity and specificity of the model. Furthermore, selecting SNPs at random from the top most significant SNPs gives models that are consistently less specific and less sensitive.
(TIF)

Figure S5 **Correlation between allele frequencies estimated with the TaqMan assay and the arrays.** The top panel shows the agreement between the allele frequencies estimated with the TaqMan assay in 688 centenarians (x-axis) and 801 centenarians of the discovery set (y-axis). The bottom panel shows the agreement between the allele frequencies estimated with the TaqMan assay in 221 controls of the NECS included in the discovery set (x-axis) and all 914 controls of the discovery set (y-axis).The difference between allele frequencies in the two groups was at most 0.04 (rs6801173). This particular SNP has substantial variability with ethnicity.
(TIF)

Figure S6 **Genes in the genetic risk models have been linked to dementia.** The networks display 42 of the 130 genes in the genetic risk model that are linked to dementia in the literature, either by functional or genetic association studies. 38 of the 42 genes are also linked to Alzheimer's disease (See Figure 6) and in red are 4 nodes that are specifically linked to dementia but not Alzheimer's disease. The nodes that are linked by an edge represent genes that are either “co-cited” (dashed lines) or “associated by expert curation” (continuous lines). The arrow head means that the associations are activation (triangle), inhibition (circle), modulation (diamond), conversion (arrow head). The node shape informs about known roles of the genes (see inset). The nodes that are singleton were linked to dementia in the literature but not together with other genes. The number of genes linked to dementia was compared to what is expected by chance using Fisher exact test, and the p-value 1.07e -6 shows that the gene set is unluckily the result of chance. (Network generated with Genomatix).
(TIF)

Figure S7 **Results of the ROC analysis in the discovery and replication sets.** Top panel: We conducted the ROC analysis using the R package “validation” for the ensemble of 281 nested models. The ensemble of model trained in the discovery set was then used to predict the outcome in the two replication sets and the predictions were assessed using ROC analysis. Bottom panel: ROC analysis of the predictions when the SNP rs2075650in TOMM40 was removed from the predictive SNPs.
(TIF)

Figure S8 **Effect of rearrangement of the top 281 SNPs and random selection of 281 SNPs from the top 1,700 most significant.** Posterior probability of exceptional longevity (EL) and average longevity (AL) (x axis) in the centenarians (red boxplots, label EL), nonagenarians-centenarians (light blue, label NN), Illumina controls (blue boxplots, label AL), in the replication set 1 (panel 1) and replication set 2 (panel 2). Panels 3 and 4 show the effect of reordering the nested models, and panels 5 and 6 show the effect of selecting a random set of 281 SNPs from the top 1,700 most significant SNPs. Numbers in parentheses denote the accuracy in each boxplot ordered from top to bottom. For example, in panel 1, 58% is the accuracy ( = specificity) in controls, 57% is the accuracy (sensitivity) in subjects of the replication set ages <103, and 71% is the accuracy (sensitivity) in the centenarians ages >102. Changing the order of the 281 SNPs decreases the difference in posterior probability of EL between centenarians and controls so that the model is less able to discriminate between centenarians and controls. The effect is even greater when the SNPs are randomly chosen from the top most significant.
(TIF)

Figure S9 **No evidence of residual stratification on individual SNP associations.** Plot of the −log10(p-value) of the 281 SNPs included in the ensemble of genetic risk models. The x-axis reports the −log10(p-value) for the unadjusted analysis, and the y-axis reports the −log10(p-value) for the analysis adjusted by the first 4 principal components. The analysis shows that there is no real change between adjusted and unadjusted analysis (correlation coefficient = 0.98.6, 99.0 and 98.2) and suggests that population stratification does not appear to confound the associations. For both analyses, we fit a logistic regression models using PLINK.
(TIF)

Figure S10 **No evidence of residual stratification on posterior probability of exceptional longevity.** **Panel A)** Plot of first two principal components (PC1 and PC2) to show the population structure in centenarians. **Panels B and C** show the principal components (PC1, and PC2, x axis) and probability of exceptional longevity (y-axis). The plot shows that the ranges of values of probability of exceptional longevity do not change in the 3 groups.
(TIF)

Figure S11 **26 genetic signatures of exceptional longevity in centenarians.** The profiles fitted in the discovery set were clustered using CAGED and hierarchical clustering and then ordered by the average genetic risk. In each plot, the x-axis reports the number of SNPs in each genetic risk model (1,…,281 SNPs), and the y-axis reports the posterior probability of exceptional longevity predicted by each model. Together, the boxplots (one for each SNP set on the x axis) display the genetic risk profiles of the centenarians in the same cluster. Numbers in parentheses are the cluster sizes (N), and the average posterior probability. Color coding represents the strength of the genetic risk to predict EL (Blue: P(EL |∑_281_)>0.95; Red: 0.5<P(EL |∑_281_)<0.95; Orange: 0.20<P(EL|∑_281_)<0.5; Green: P(EL|∑_281_)<0.2). Only clusters with 8 or more centenarians are included and describe 90% of all cases in the discovery set.
(TIF)

Figure S12 **Clusters of profiles predicted in the replication set comprising the ELIXIR subjects and the additional set of 60 centenarians from the NECS.** Only clusters with 8 or more centenarians are included. Several of the signatures discovered in the replication set match signatures in the discovery set: The pattern of R1 matches C1, R2 matches C2, R4 matches C6, R5 matches C11, R8 matches C19, R15 matches C26. The profiles were generated using the genetic risk models trained in the discovery set. The profiles were then clustered using CAGED and hierarchical clustering and then ranked by the average posterior probability of exceptional longevity per cluster.
(TIF)

Figure S13 **Clusters of profiles of the controls in the discovery set.** Genetic signatures in 845 controls subjects of the discovery set. Numbers in parentheses are the cluster sizes (N), and the average posterior probability of exceptional longevity per cluster. Color coding represents the strength of the genetic risk to predict EL (Blue: P(EL|∑_281_)>0.95, Red: 0.5<P(EL|∑_281_)<0.95; Orange: 0.20<P(EL|∑_281_)<0.5; Green: P(EL|∑_281_)<0.2).
(TIF)

Figure S14 **Summary of genetic signatures of exceptional longevity in the centenarians of the discovery set and 4118 controls.** We used the nested genetic risk models trained in the discovery set to compute the genetic profiles of all controls, and clustered the profiles using the same analytic strategy. The cluster analysis grouped subjects in 254 clusters of 7 or more, while the remaining subjects had more sporadic signatures. The pie charts display the distribution of all genetic signatures in the 801 centenarians of the discovery set (left) and the 4118 controls (right). The slices are color coded as in the previous figures (Blue: p(EL|∑_281_)>0.95; Red: 0.70<P(EL|∑_281_)<0.95; Brown: 0.5<P(EL|∑_281_)<0.7; Orange: 0.17<P(EL|∑_281_)<0.50; Green P(EL|∑_281_)<0.17). The label P(E) denotes p(EL|∑_281_). Note the almost lack of “blue” and the dominance of “green” and “orange” signatures in the control set compared to the centenarian set.
(TIF)

Figure S15 **Signatures with random profiles.** To compare the results from cluster analysis of genetic risk profiles and derived signatures against random results, we randomly selected 300 SNPs from the list of analyzed SNPs, we generated a set of nested genetic risk models using the procedure described in the manuscript and then we tried to cluster the genetic risk profiles. We repeated this analysis a few times, and consistently showed that sensitivity and specificity in the replication set were 0.5 (pure chance), and when we attempted to cluster the genetic risk profiles the analysis produced many smaller clusters (average size 3 profile per clusters compared to 15 profiles per cluster in the signatures generated in the manuscript), many profiles that could not be clustered at all, and those profiles that could be clustered more effectively were showing random variability around 0.5.
(TIF)

Figure S16 **Age distribution of centenarians in the 26 genetic signatures in the discovery set and in 15 signatures of the merged replication sets.** The boxplots were generated with the R package, and the box displays the ages at death between the 25^th^ and 75^th^ percentile, with median age depicted as the middle bar. The whiskers extend to the most extreme data point which is no more than 1.5 times the interquartile range from the box. The boxplots are ordered by predictive accuracy of the genetic risk models within clusters. (Blue: P(EL|∑_281_)>0.95,; Red: 0.5<P(EL|∑_281_)<0.95; Orange: 0.20<P(EL|∑_281_)<0.5; Green: P(EL|∑_281_)<0.2). The most predictive cluster (C1) is associated with the longest median survival, and other genetic signatures are characterized by different survivals as well.
(TIF)

Figure S17 **Distributions of age of onset to cardiovascular disease (CVD), pulmonary disease (CPD), macular degeneration (MD) and hypertension between centenarians with different genetic signatures.** The x-axis reports age of events, and the y-axis reports the event-free survival distribution. Only subjects with events were included in the analysis. The caption below each plot indicates the disease and the p-value to test significance differences using the log-rank test. Median ages of onsets are in the insets. Subjects in cluster C1 had a significant delay in the onset of dementia and stroke, compared to other clusters. They also delayed onset of cancer compared to centenarians with signatures C2, C3 and C5, but not differently from centenarians with signature C6, and delayed cardiovascular disease compared to centenarians with other signatures but not differently from centenarians with signature C3. Ages of onset of other diseases also differ between other clusters.
(TIF)

Figure S18 **Pedigrees of 2 centenarians in a cluster showing no prediction for exceptional longevity (C26).** The two pedigrees show examples of familial longevity although the genetic risk profiles of the two centenarian probands (red arrows) show no enrichment of longevity associated variants. This could indicate that such families have more private or rare variants not captured by either the genotyping or the model.
(TIF)

Figure S19 **Predictive value of the SNP rs2075650 in TOMM40/APOE in the discovery set.** The table reports the posterior probability of exceptional and average longevity for different genotypes of rs2075650. The ROC analysis shows that this SNP alone cannot optimize the trade off between sensitivity and specificity. The area under the curve is 0.62 compared to 0.95 when 281 SNPs are used in the model (Figure S7, top, left panel). Note that some threshold on the posterior probability can produce an accuracy that is worse than random classification.
(TIF)

Figure S20 **Plot of allele frequencies in 32 subjects genotyped with both the Humanhap CNV370 Illumina array (x axis) and HumanHap 610-Quad Illumina array (y-axis).** Dots in the boundaries of the figure represent inconsistent SNPs between arrays. Only SNPs that had CR>97% are included.
(TIF)

Figure S21 **Agreement of allele frequencies in different SNP arrays.** **Panel** A) shows the plot of allele frequencies in 573 centenarians genotyped with array HumanHap370 (x-axis) and 168 centenarians genotyped with the HumanHap 1 M (y-axis). Panel B) shows the allele frequency in 151 controls typed with array HumanHap330 (x-axis) and 863 with HumanHap 550 (y-axis). Panel C shows C) shows the allele frequency in 241 controls typed with array HumanHap370 (x-axis) and 863 with HumanHap 550 (y-axis).
(TIF)

Table S1 **List of 281 SNPs included in the genetic risk model.** This is an excel file with 3 worksheets. “README” worksheet describes the column contents; “281 SNPs” worksheet describes the list of 281 SNPs used in the ensemble genetic risk models. This includes details about call rate by array type and phenotype, details of QC, statistical analysis. “Functional annotation” worksheet includes functional annotation of the 281 SNPs.
(XLS)

Table S2 **Disease prevalence in clusters of centenarians with different genetic signatures.** Cardiovascular disease defined as angina, congestive heart failure, peripheral circulatory disease or myocardial infarction; pulmonary disease is asthma, chronic bronchitis or emphysema; hypertension: systolic blood pressure >140 mm Hg and/or diastolic blood pressure >90 mm Hg or on medication for HTN.
(DOCX)

Table S3 **Rate of disease associated variants carried by centenarians and controls, and p-value from Student's T test.** Risk alleles were derived from the GWAS catalogue at the NHGRI (downloaded in April 2011) and the Human Genome Mutation Database. The boxplots displays the rate of risk alleles carried by centenarians (blue) and controls (red). The disease described are: lupus, cholesterol level (Chol), macular degeneration (MD), Parkinson's Disease (PD), Chron's disease (chr), diabetes (diab), cardiovascular disease (CVD), cance (canc)r, Alzheimer's (AD), GWAS.pt is the group of alleles related to personality disorders that were found in GWAS, gwas.qt is the group of alleles related to QTL from GWASs and include cholesterol, BMI, obesity etc, and GWAS.cc is the group of risk alleles found from case/control GWASs so include for example cancer, PD, MD etc, cod is for coding variants from the HGMD, and all is the full set of 1214 variants.
(DOCX)

Table S4 **List of disease associated SNPs that showed significant differences in the discovery sets.** Highlighted in grey are the SNPs with risk alleles that are less common in centenarians. Some SNPs had unreported risk alleles in the original publications that are denoted with a question mark.
(DOCX)
