# Supplementary figures and images for: Genetic Signatures of Exceptional Longevity in Humans
Source: PLoS One. 2012 Jan 18;7(1):e29848. doi: 10.1371/journal.pone.0029848 (PMC3261167; doi:10.1371/journal.pone.0029848)

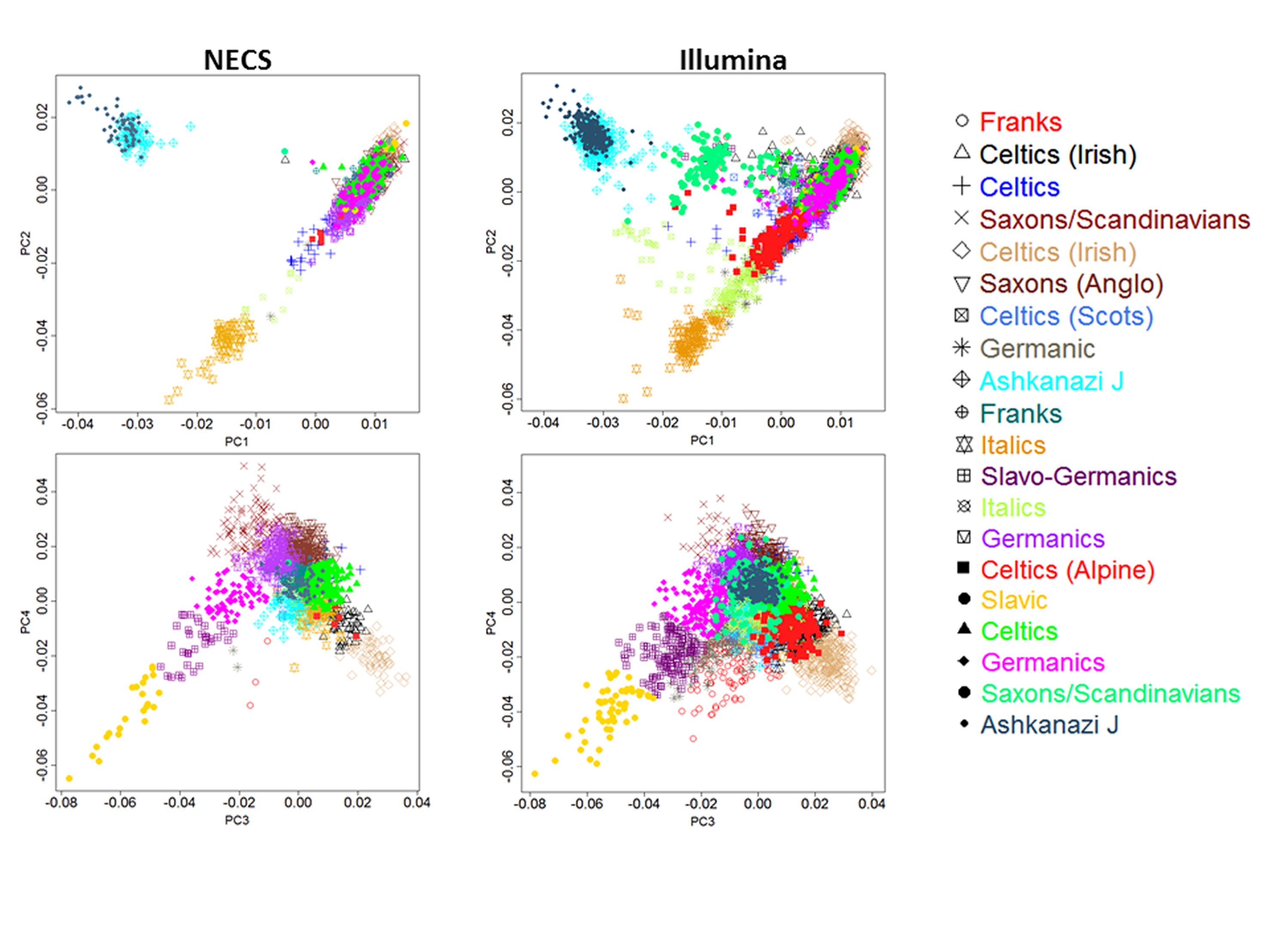

Supplement: Figure S1 — Population structure of centenarians and controls. Scatter plot of principal components 1 and 2 (PC1 and 2 PC2, top panels), and principal components 3 and 4 (PC3 and PC4, bottom panels) in subjects from the NECS (left) and Illumina database (right) that were estimated using genome wide data. We labeled the clusters by ethnicity using the information about mother tongue and place of birth of NECS subjects and their parents. Note that some of the European ethnic groups in controls (NECS and Illumina) are not represented in NECS cases, for example Italics (⊗ green), Saxon/Scandinavia (• green), Celtics/Alpine (▪ red), and Franks (, red). (TIF) [file pone.0029848.s001.tif]

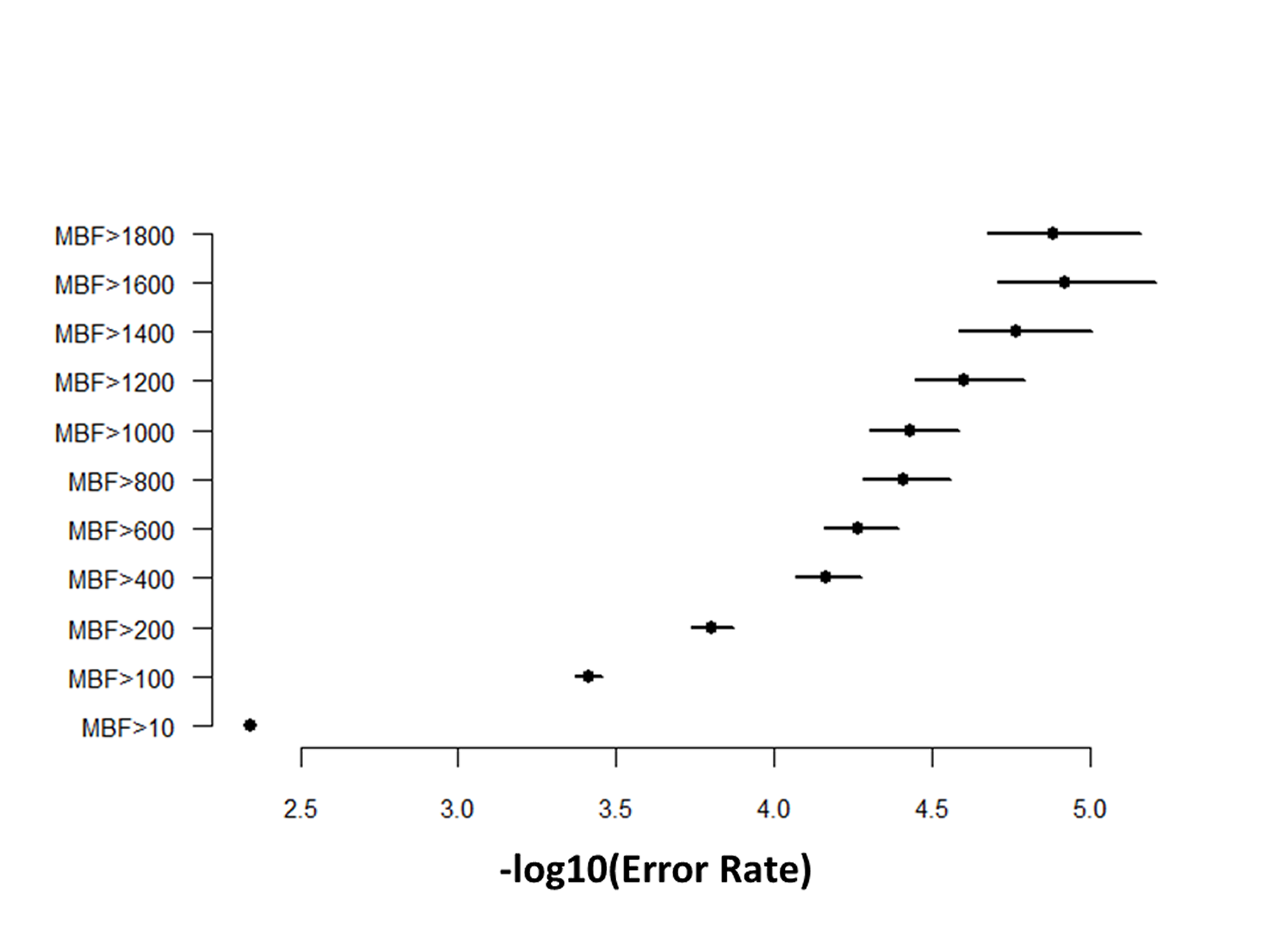

Supplement: Figure S2 — Error rate in log10 scale of the Bayes rule for different thresholds of the MBF. The x axes reports the estimate of the −log10(error rate) and 95% credible intervals that were estimated using a Beta distribution in 1,000,000 simulations per threshold on the MBF (y-axis). The MBF is the maximum Bayes Factor computed to test the association of each SNP in 4 genetic models (genotypic, allelic, dominant, recessive). The genotype data were generated with allele frequencies varying uniformly between 0.05 and 0.5 and assuming HWE. The analysis suggests that a MBF>1,400 determines an error rate of approximately 1 to 2 errors per 100,000 tested association (−log10(2/100,000) = 4.7)), and a MBF>100 determines an error rate of approximately 4 errors per 100,000 tested association (−log10(4/100,000) = 3.4). Note that this analysis includes the additional costs of searching for 4 genetic models. (TIF) [file pone.0029848.s002.tif]

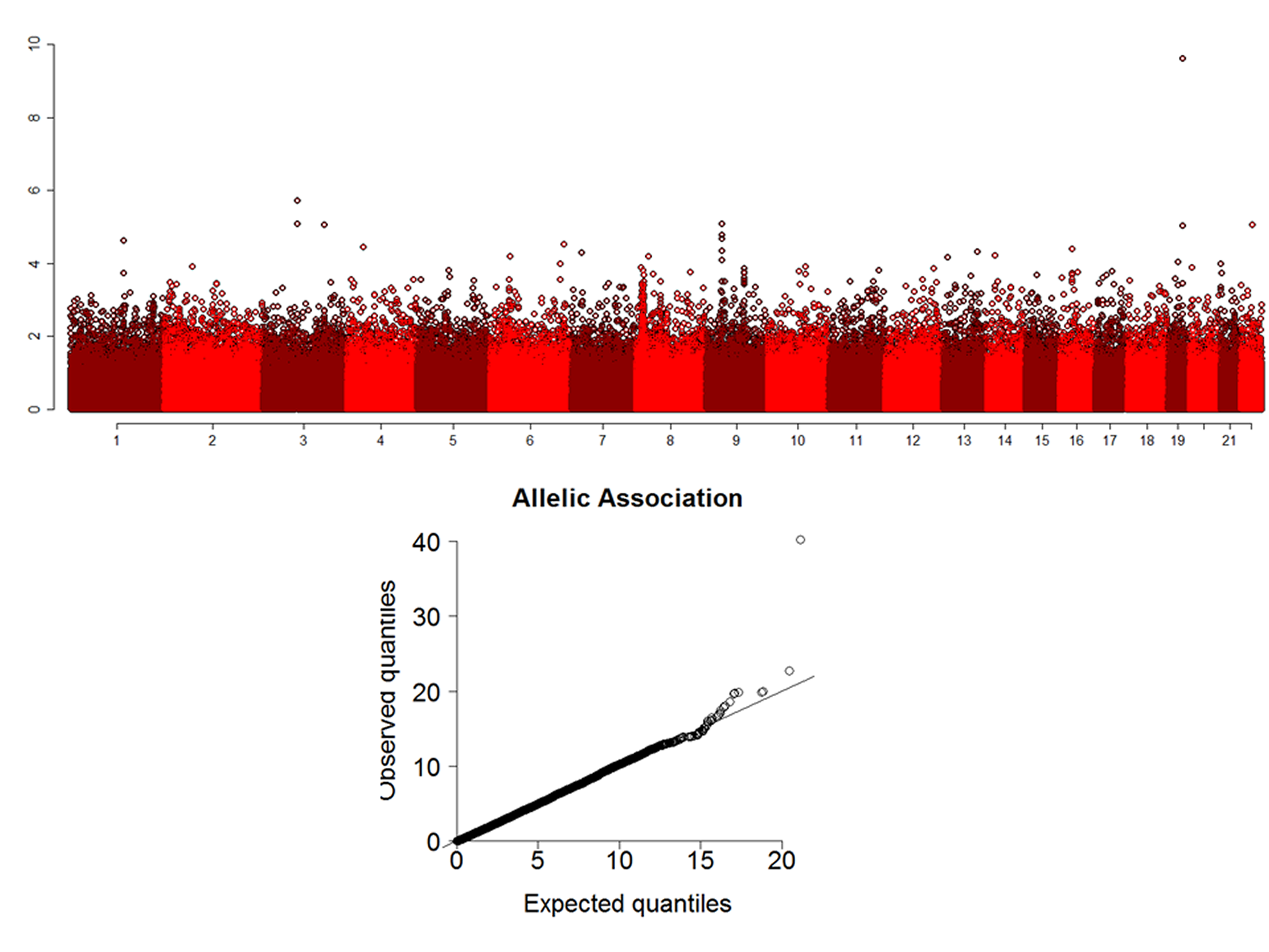

Supplement: Figure S3 — Manhattan plot and QQ-plot for the allelic association tested using a traditional frequentist approach. The Manhattan plot shows the −log10(p-value) for the 1 degree of freedom test Chi-square test. The QQ-plot displays the observed quantiles of the 1 degree of freedom test Chi-square test versus the expected quantiles. (TIF) [file pone.0029848.s003.tif]

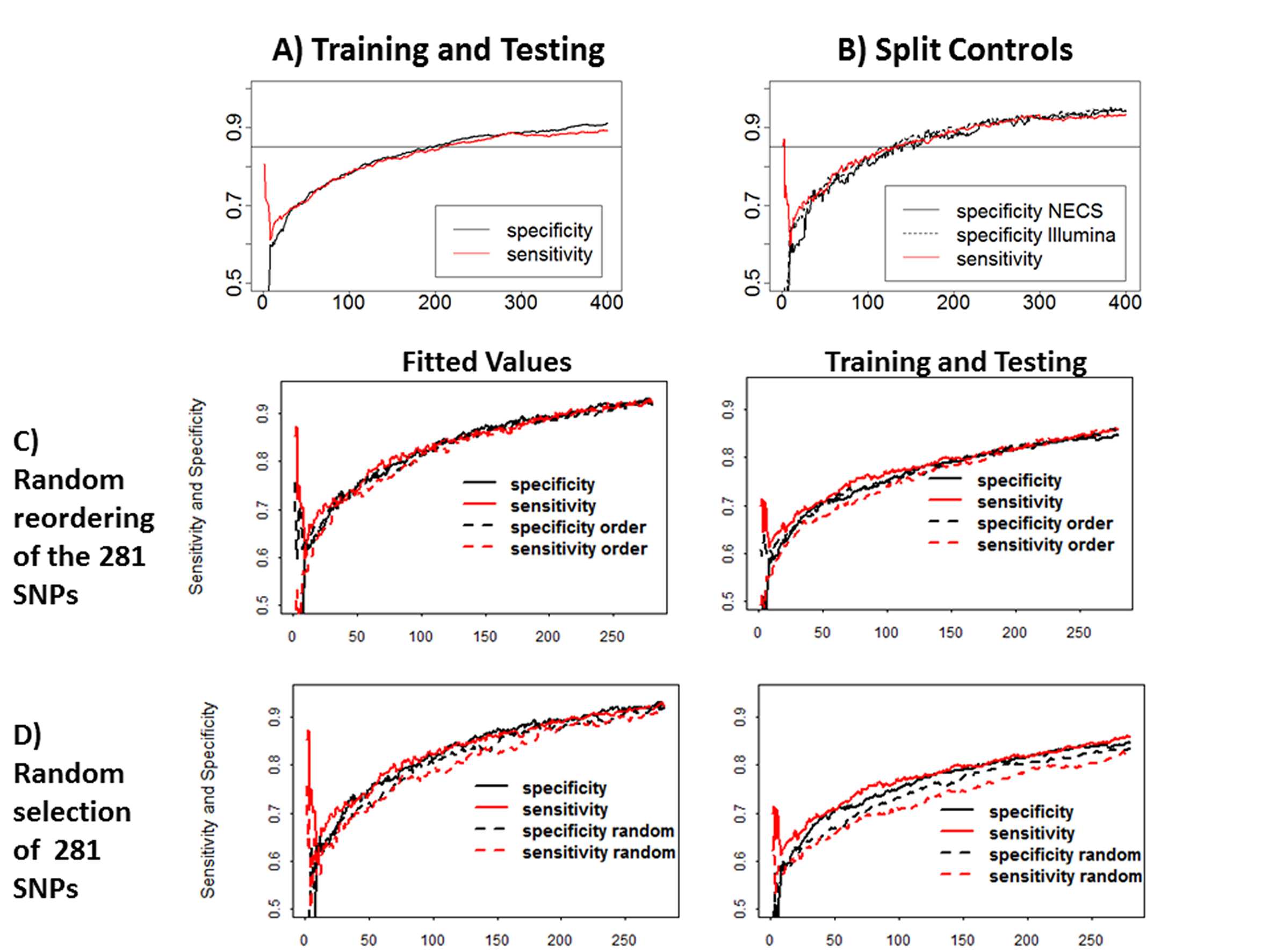

Supplement: Figure S4 — Effect of sampling variability, and SNP ordering on the sensitivity and specificity of the model. Panel A) displays the average sensitivity and specificity of 400 nested models in 1000 resampled sets. 1000 training and test sets were randomly resampled from the discovery set and each training set was used to estimate the Bayesian classification rule that was tested in the test set. The plot displays the average sensitivity and specificity (y-axis) versus number of SNPs (x-axis). The sensitivity is the proportion of centenarians with posterior probability of exceptional longevity>posterior probability of average longevity and the specificity is the proportion of controls with posterior probability of exceptional longevity<posterior probability of average longevity. The mean number of SNPs in which the absolute difference between sensitivity and specificity was <0.02 and accuracy was >85% was 281. Panel B) displays the specificity for the two types of controls in the discovery set (NECS referent subjects: continuous line; Illumina controls: dashed lines) and shows that there is no difference between the two control sets. Panel C) describes the effect of re-ordering the 281 SNPs. Patterns of sensitivity and specificity using the discovery set (left), and randomly generated validation sets (right) when the top 281 SNPs were randomly entered into the nested models (continuous lines: SNPs are ordered by MBF; dashed lines: the same 281 SNPs are randomly arranged). Panel D) describes the effect of random selection on sensitivity and specificity of the nested models. Patterns of sensitivity and specificity using the discovery set (left), and randomly generated validation sets (right) when 281 SNPs were randomly chosen from the top 1,700 most significant SNPs. (continuous lines: SNPs are ordered by MBF; dashed lines: 281 SNPs are randomly selected from the 1700 most significant). The analysis shows that changing the order affects sensitivity and specificity of the model. Furt [file pone.0029848.s004.tif]

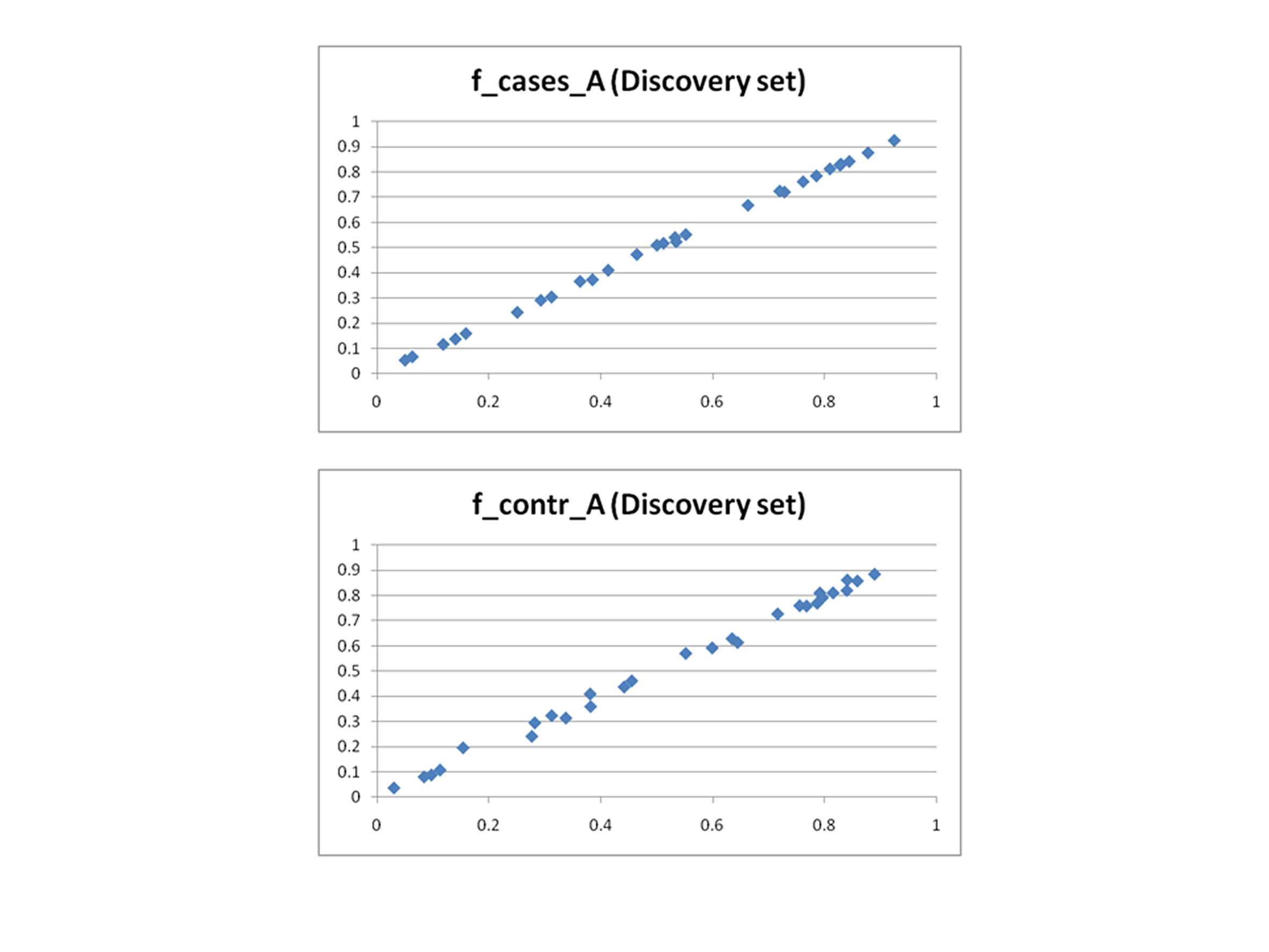

Supplement: Figure S5 — Correlation between allele frequencies estimated with the TaqMan assay and the arrays. The top panel shows the agreement between the allele frequencies estimated with the TaqMan assay in 688 centenarians (x-axis) and 801 centenarians of the discovery set (y-axis). The bottom panel shows the agreement between the allele frequencies estimated with the TaqMan assay in 221 controls of the NECS included in the discovery set (x-axis) and all 914 controls of the discovery set (y-axis).The difference between allele frequencies in the two groups was at most 0.04 (rs6801173). This particular SNP has substantial variability with ethnicity. (TIF) [file pone.0029848.s005.tif]

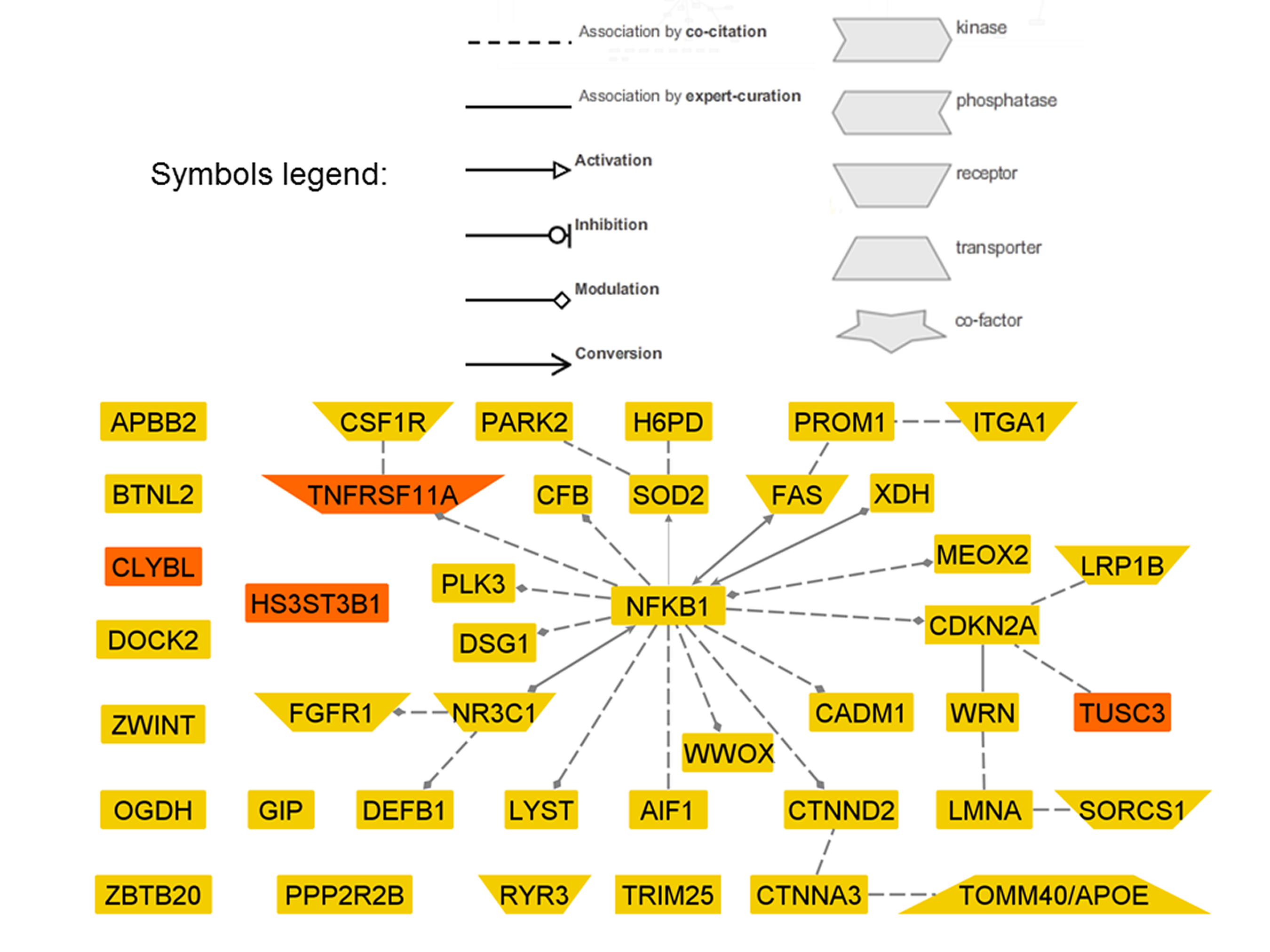

Supplement: Figure S6 — Genes in the genetic risk models have been linked to dementia. The networks display 42 of the 130 genes in the genetic risk model that are linked to dementia in the literature, either by functional or genetic association studies. 38 of the 42 genes are also linked to Alzheimer's disease (See Figure 6) and in red are 4 nodes that are specifically linked to dementia but not Alzheimer's disease. The nodes that are linked by an edge represent genes that are either “co-cited” (dashed lines) or “associated by expert curation” (continuous lines). The arrow head means that the associations are activation (triangle), inhibition (circle), modulation (diamond), conversion (arrow head). The node shape informs about known roles of the genes (see inset). The nodes that are singleton were linked to dementia in the literature but not together with other genes. The number of genes linked to dementia was compared to what is expected by chance using Fisher exact test, and the p-value 1.07e -6 shows that the gene set is unluckily the result of chance. (Network generated with Genomatix). (TIF) [file pone.0029848.s006.tif]

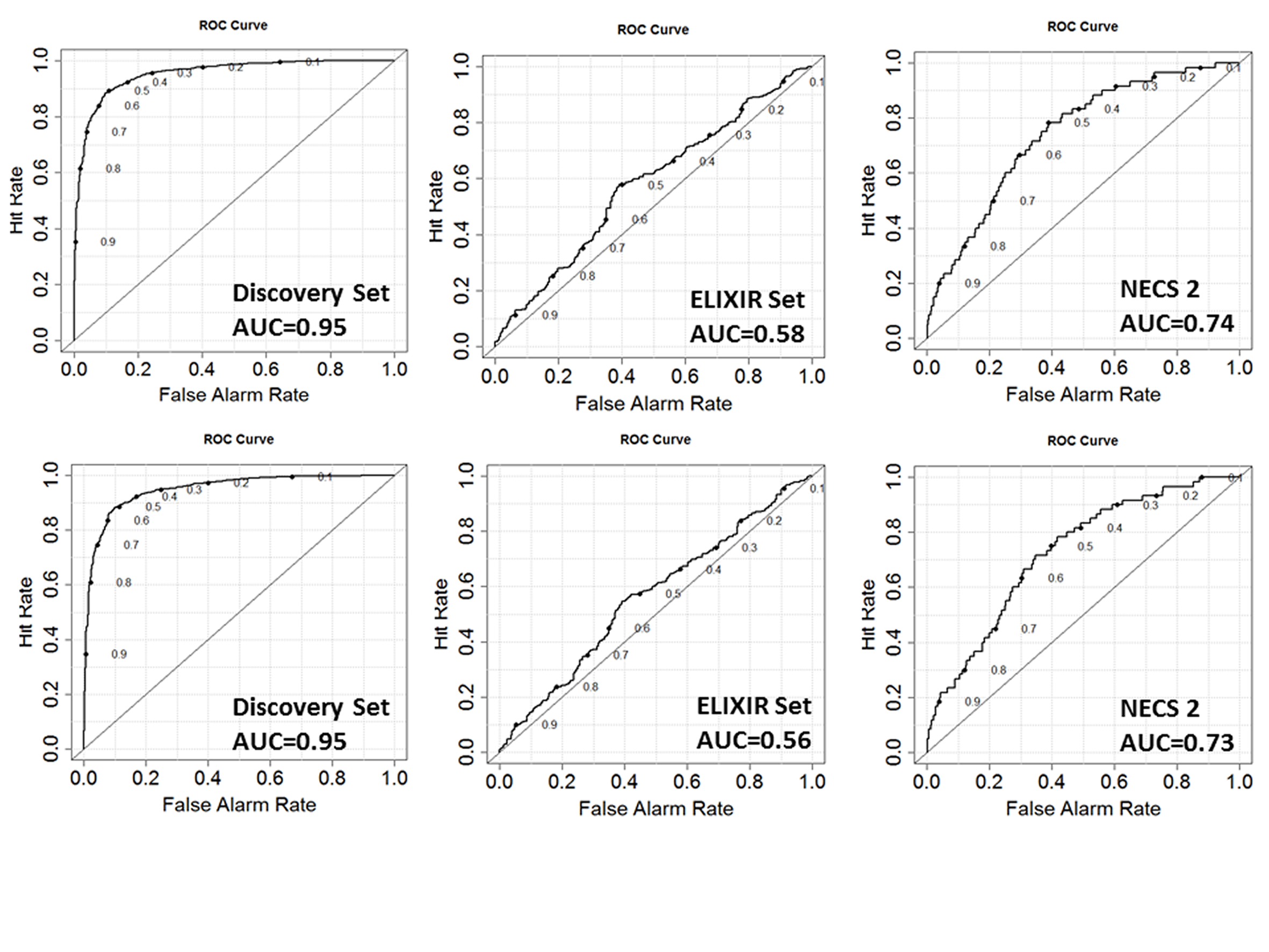

Supplement: Figure S7 — Results of the ROC analysis in the discovery and replication sets. Top panel: We conducted the ROC analysis using the R package “validation” for the ensemble of 281 nested models. The ensemble of model trained in the discovery set was then used to predict the outcome in the two replication sets and the predictions were assessed using ROC analysis. Bottom panel: ROC analysis of the predictions when the SNP rs2075650in TOMM40 was removed from the predictive SNPs. (TIF) [file pone.0029848.s007.tif]

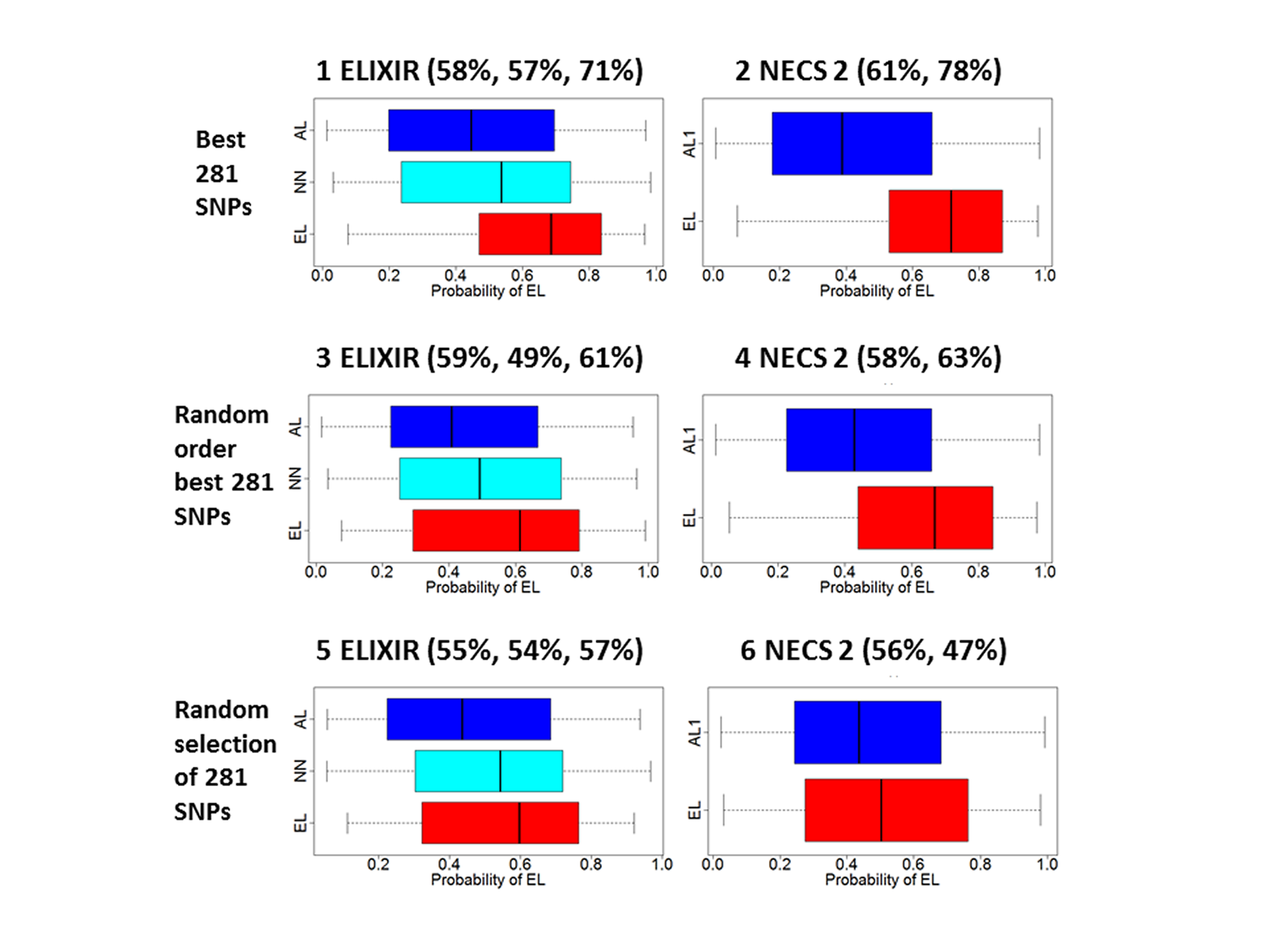

Supplement: Figure S8 — Effect of rearrangement of the top 281 SNPs and random selection of 281 SNPs from the top 1,700 most significant. Posterior probability of exceptional longevity (EL) and average longevity (AL) (x axis) in the centenarians (red boxplots, label EL), nonagenarians-centenarians (light blue, label NN), Illumina controls (blue boxplots, label AL), in the replication set 1 (panel 1) and replication set 2 (panel 2). Panels 3 and 4 show the effect of reordering the nested models, and panels 5 and 6 show the effect of selecting a random set of 281 SNPs from the top 1,700 most significant SNPs. Numbers in parentheses denote the accuracy in each boxplot ordered from top to bottom. For example, in panel 1, 58% is the accuracy ( = specificity) in controls, 57% is the accuracy (sensitivity) in subjects of the replication set ages <103, and 71% is the accuracy (sensitivity) in the centenarians ages >102. Changing the order of the 281 SNPs decreases the difference in posterior probability of EL between centenarians and controls so that the model is less able to discriminate between centenarians and controls. The effect is even greater when the SNPs are randomly chosen from the top most significant. (TIF) [file pone.0029848.s008.tif]

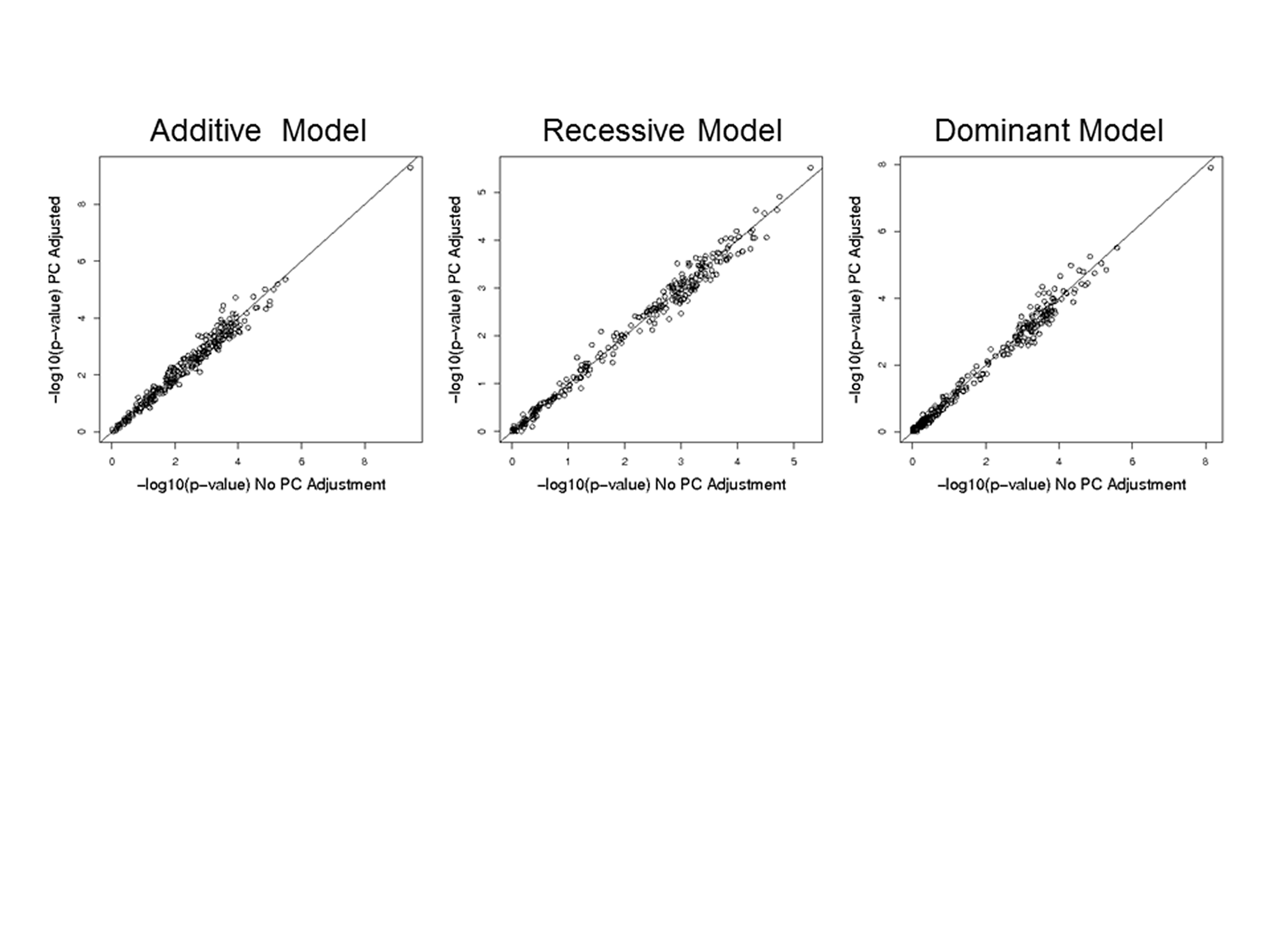

Supplement: Figure S9 — No evidence of residual stratification on individual SNP associations. Plot of the −log10(p-value) of the 281 SNPs included in the ensemble of genetic risk models. The x-axis reports the −log10(p-value) for the unadjusted analysis, and the y-axis reports the −log10(p-value) for the analysis adjusted by the first 4 principal components. The analysis shows that there is no real change between adjusted and unadjusted analysis (correlation coefficient = 0.98.6, 99.0 and 98.2) and suggests that population stratification does not appear to confound the associations. For both analyses, we fit a logistic regression models using PLINK. (TIF) [file pone.0029848.s009.tif]

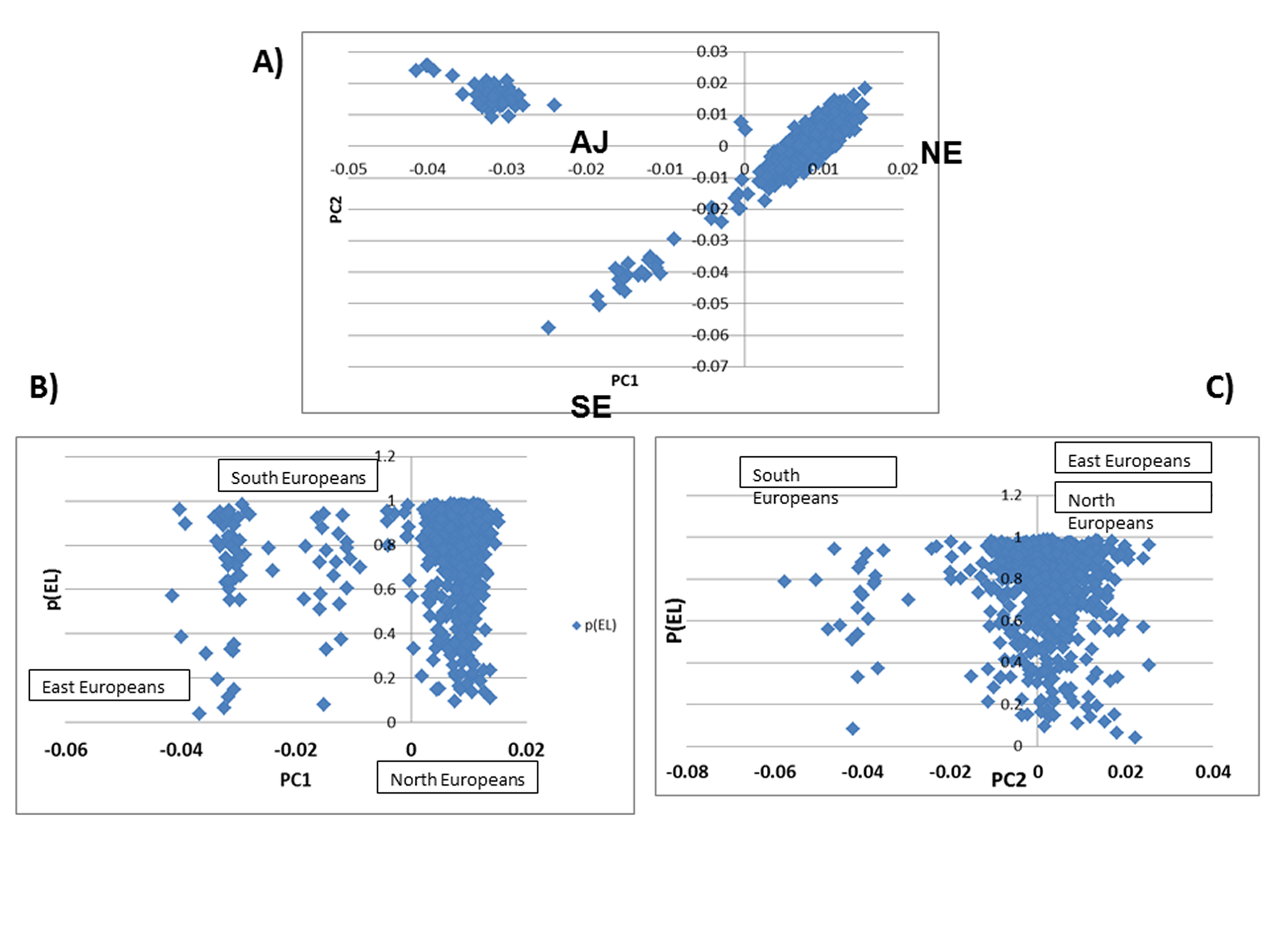

Supplement: Figure S10 — No evidence of residual stratification on posterior probability of exceptional longevity. Panel A) Plot of first two principal components (PC1 and PC2) to show the population structure in centenarians. Panels B and C show the principal components (PC1, and PC2, x axis) and probability of exceptional longevity (y-axis). The plot shows that the ranges of values of probability of exceptional longevity do not change in the 3 groups. (TIF) [file pone.0029848.s010.tif]

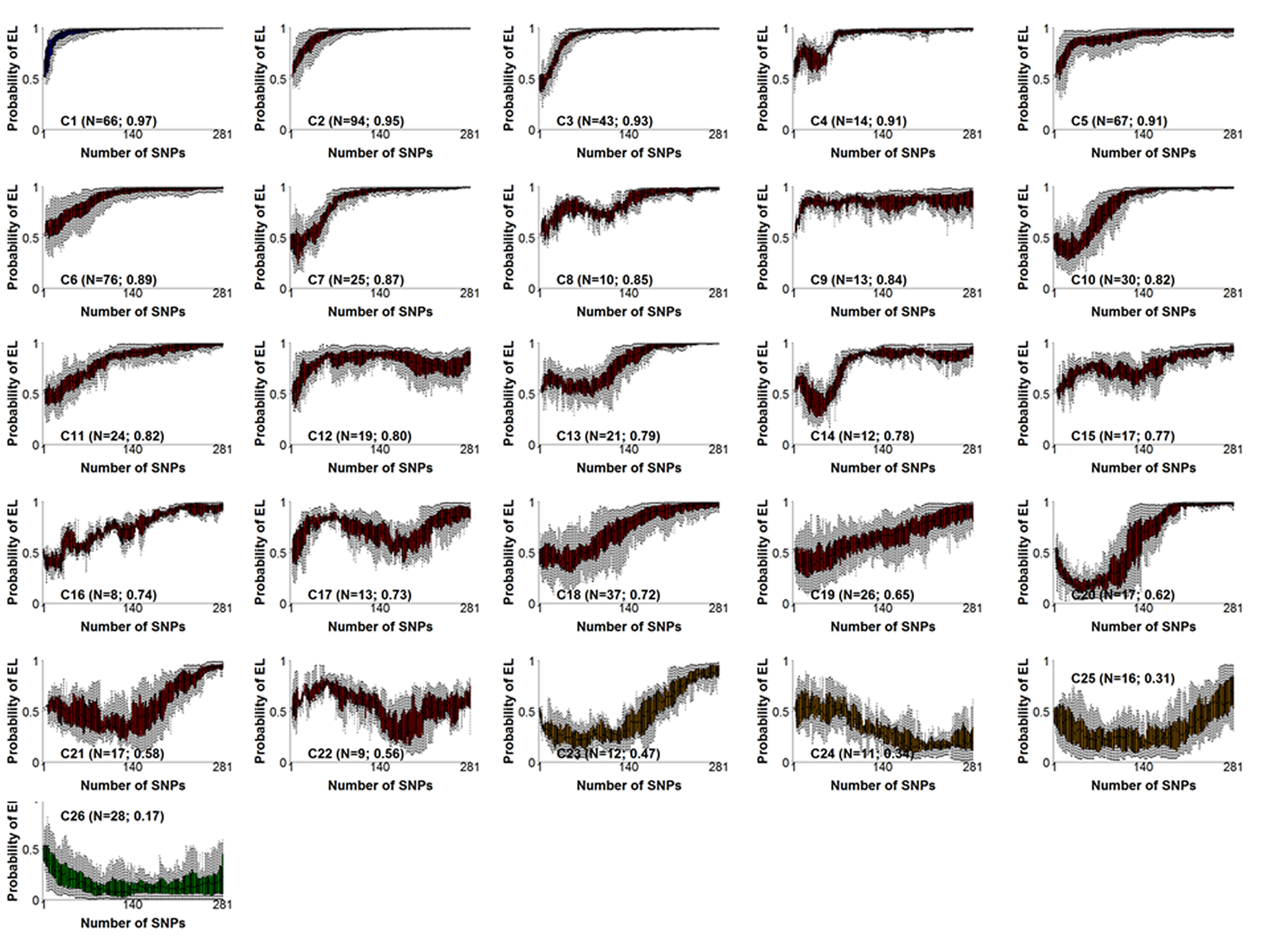

Supplement: Figure S11 — 26 genetic signatures of exceptional longevity in centenarians. The profiles fitted in the discovery set were clustered using CAGED and hierarchical clustering and then ordered by the average genetic risk. In each plot, the x-axis reports the number of SNPs in each genetic risk model (1,…,281 SNPs), and the y-axis reports the posterior probability of exceptional longevity predicted by each model. Together, the boxplots (one for each SNP set on the x axis) display the genetic risk profiles of the centenarians in the same cluster. Numbers in parentheses are the cluster sizes (N), and the average posterior probability. Color coding represents the strength of the genetic risk to predict EL (Blue: P(EL |∑281)>0.95; Red: 0.5<P(EL |∑281)<0.95; Orange: 0.20<P(EL|∑281)<0.5; Green: P(EL|∑281)<0.2). Only clusters with 8 or more centenarians are included and describe 90% of all cases in the discovery set. (TIF) [file pone.0029848.s011.tif]

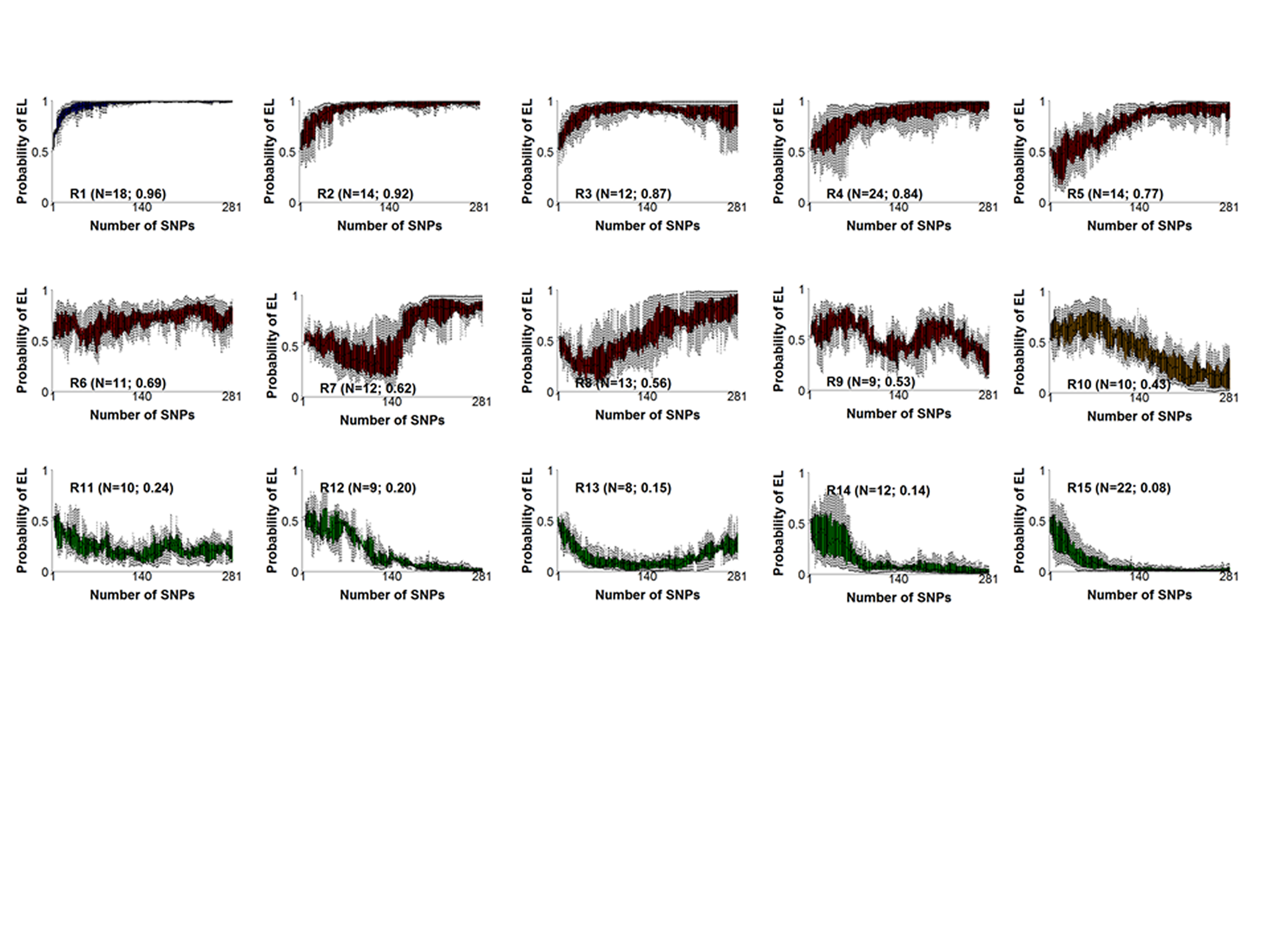

Supplement: Figure S12 — Clusters of profiles predicted in the replication set comprising the ELIXIR subjects and the additional set of 60 centenarians from the NECS. Only clusters with 8 or more centenarians are included. Several of the signatures discovered in the replication set match signatures in the discovery set: The pattern of R1 matches C1, R2 matches C2, R4 matches C6, R5 matches C11, R8 matches C19, R15 matches C26. The profiles were generated using the genetic risk models trained in the discovery set. The profiles were then clustered using CAGED and hierarchical clustering and then ranked by the average posterior probability of exceptional longevity per cluster. (TIF) [file pone.0029848.s012.tif]

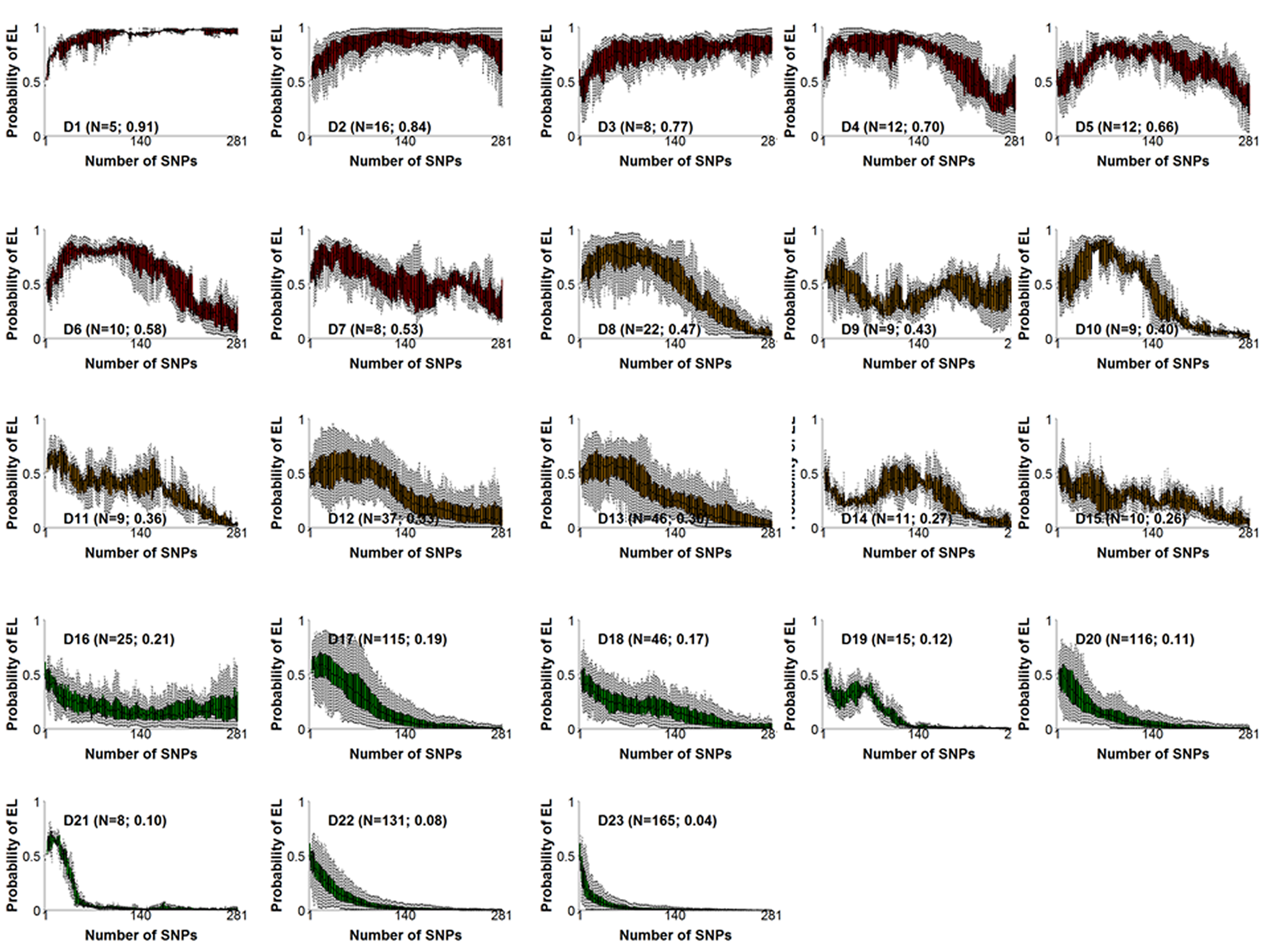

Supplement: Figure S13 — Clusters of profiles of the controls in the discovery set. Genetic signatures in 845 controls subjects of the discovery set. Numbers in parentheses are the cluster sizes (N), and the average posterior probability of exceptional longevity per cluster. Color coding represents the strength of the genetic risk to predict EL (Blue: P(EL|∑281)>0.95, Red: 0.5<P(EL|∑281)<0.95; Orange: 0.20<P(EL|∑281)<0.5; Green: P(EL|∑281)<0.2). (TIF) [file pone.0029848.s013.tif]

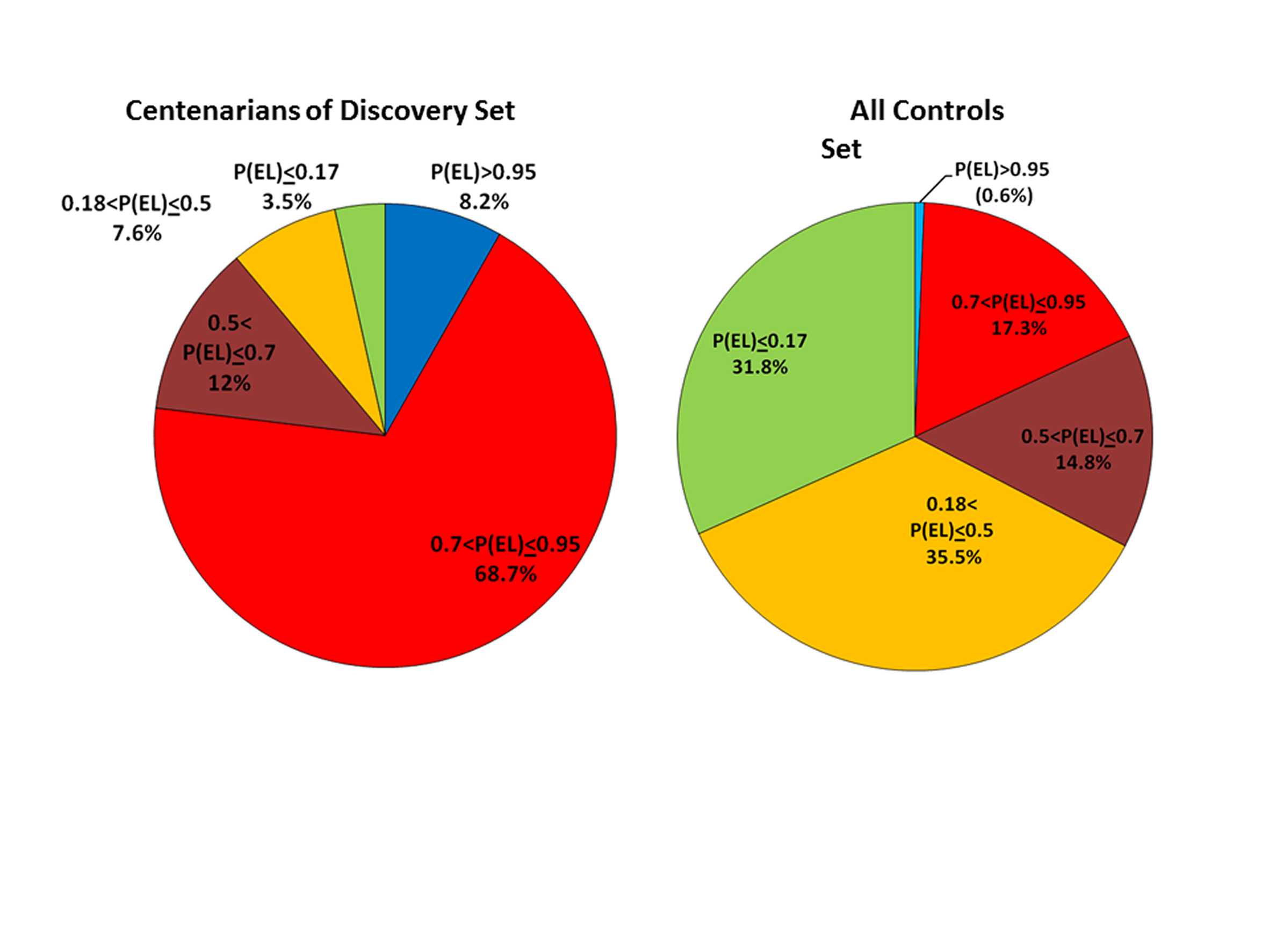

Supplement: Figure S14 — Summary of genetic signatures of exceptional longevity in the centenarians of the discovery set and 4118 controls. We used the nested genetic risk models trained in the discovery set to compute the genetic profiles of all controls, and clustered the profiles using the same analytic strategy. The cluster analysis grouped subjects in 254 clusters of 7 or more, while the remaining subjects had more sporadic signatures. The pie charts display the distribution of all genetic signatures in the 801 centenarians of the discovery set (left) and the 4118 controls (right). The slices are color coded as in the previous figures (Blue: p(EL|∑281)>0.95; Red: 0.70<P(EL|∑281)<0.95; Brown: 0.5<P(EL|∑281)<0.7; Orange: 0.17<P(EL|∑281)<0.50; Green P(EL|∑281)<0.17). The label P(E) denotes p(EL|∑281). Note the almost lack of “blue” and the dominance of “green” and “orange” signatures in the control set compared to the centenarian set. (TIF) [file pone.0029848.s014.tif]

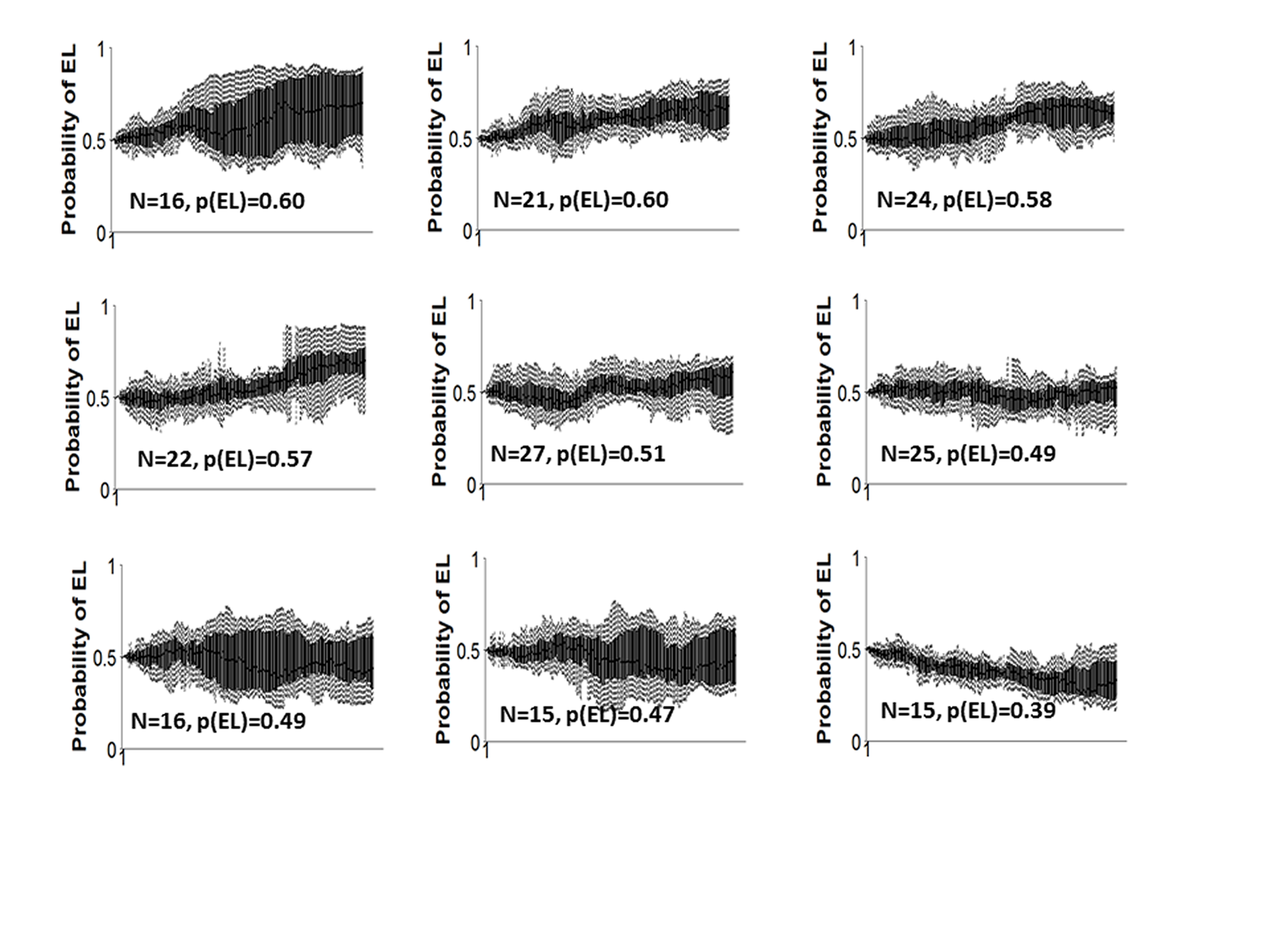

Supplement: Figure S15 — Signatures with random profiles. To compare the results from cluster analysis of genetic risk profiles and derived signatures against random results, we randomly selected 300 SNPs from the list of analyzed SNPs, we generated a set of nested genetic risk models using the procedure described in the manuscript and then we tried to cluster the genetic risk profiles. We repeated this analysis a few times, and consistently showed that sensitivity and specificity in the replication set were 0.5 (pure chance), and when we attempted to cluster the genetic risk profiles the analysis produced many smaller clusters (average size 3 profile per clusters compared to 15 profiles per cluster in the signatures generated in the manuscript), many profiles that could not be clustered at all, and those profiles that could be clustered more effectively were showing random variability around 0.5. (TIF) [file pone.0029848.s015.tif]

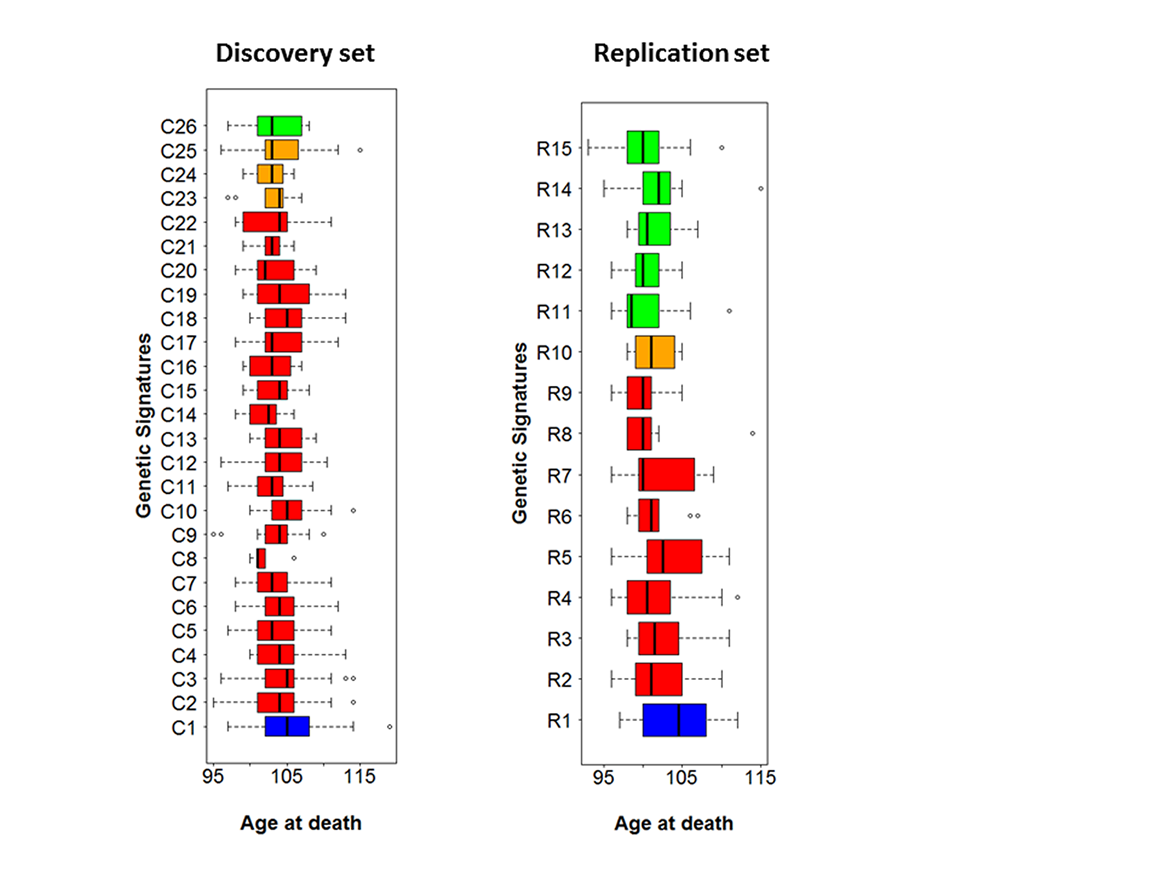

Supplement: Figure S16 — Age distribution of centenarians in the 26 genetic signatures in the discovery set and in 15 signatures of the merged replication sets. The boxplots were generated with the R package, and the box displays the ages at death between the 25th and 75th percentile, with median age depicted as the middle bar. The whiskers extend to the most extreme data point which is no more than 1.5 times the interquartile range from the box. The boxplots are ordered by predictive accuracy of the genetic risk models within clusters. (Blue: P(EL|∑281)>0.95,; Red: 0.5<P(EL|∑281)<0.95; Orange: 0.20<P(EL|∑281)<0.5; Green: P(EL|∑281)<0.2). The most predictive cluster (C1) is associated with the longest median survival, and other genetic signatures are characterized by different survivals as well. (TIF) [file pone.0029848.s016.tif]

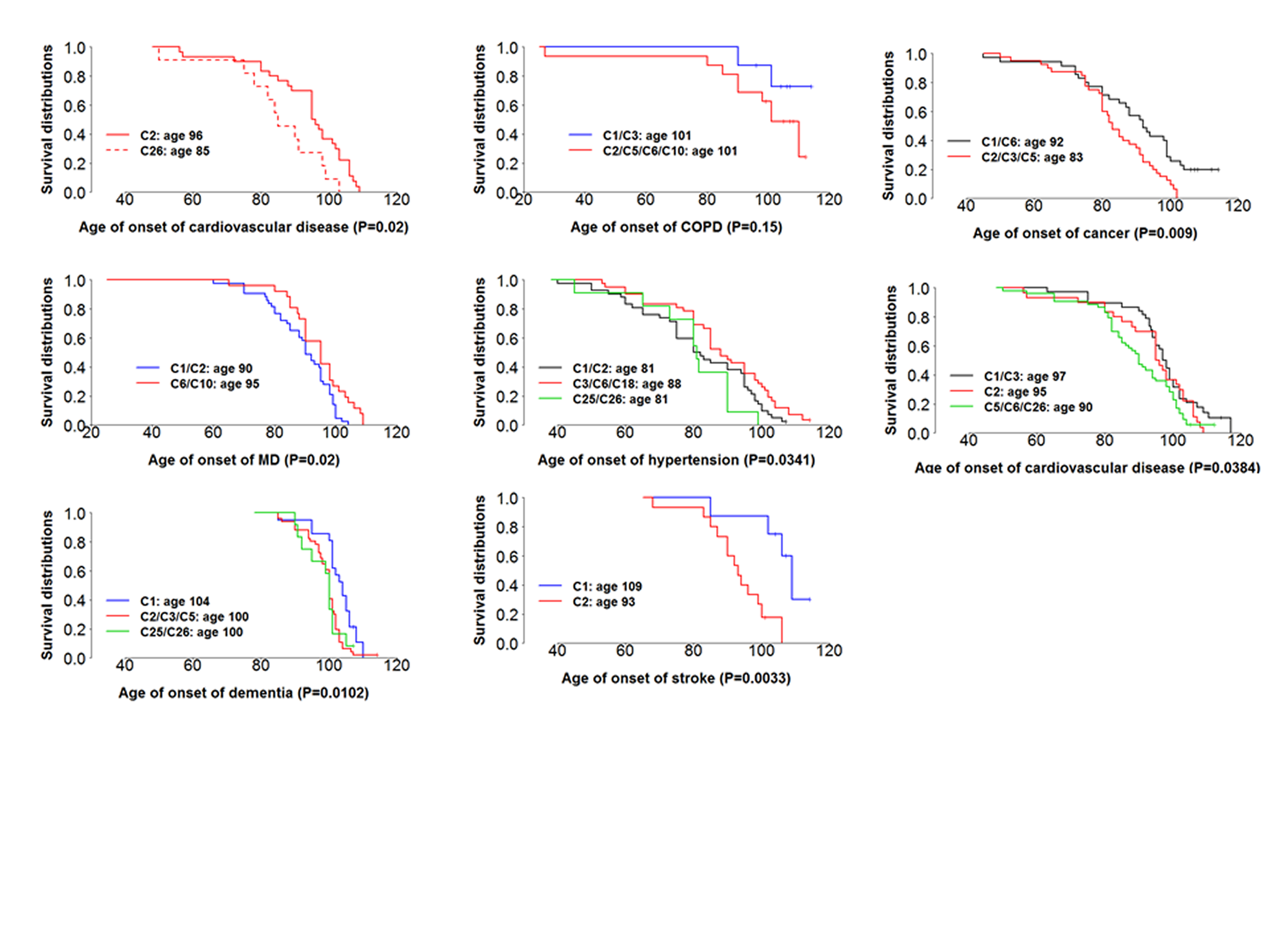

Supplement: Figure S17 — Distributions of age of onset to cardiovascular disease (CVD), pulmonary disease (CPD), macular degeneration (MD) and hypertension between centenarians with different genetic signatures. The x-axis reports age of events, and the y-axis reports the event-free survival distribution. Only subjects with events were included in the analysis. The caption below each plot indicates the disease and the p-value to test significance differences using the log-rank test. Median ages of onsets are in the insets. Subjects in cluster C1 had a significant delay in the onset of dementia and stroke, compared to other clusters. They also delayed onset of cancer compared to centenarians with signatures C2, C3 and C5, but not differently from centenarians with signature C6, and delayed cardiovascular disease compared to centenarians with other signatures but not differently from centenarians with signature C3. Ages of onset of other diseases also differ between other clusters. (TIF) [file pone.0029848.s017.tif]

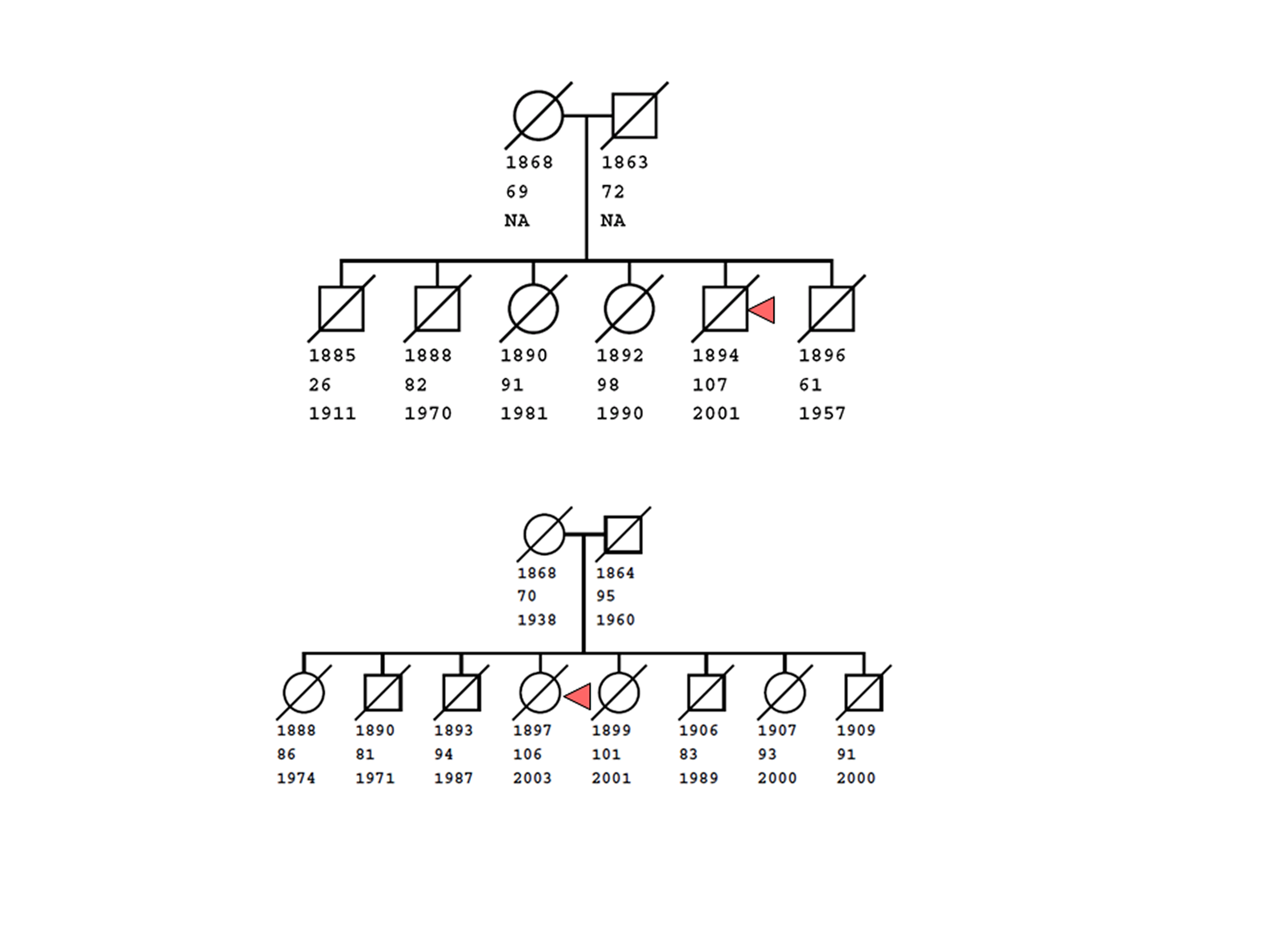

Supplement: Figure S18 — Pedigrees of 2 centenarians in a cluster showing no prediction for exceptional longevity (C26). The two pedigrees show examples of familial longevity although the genetic risk profiles of the two centenarian probands (red arrows) show no enrichment of longevity associated variants. This could indicate that such families have more private or rare variants not captured by either the genotyping or the model. (TIF) [file pone.0029848.s018.tif]

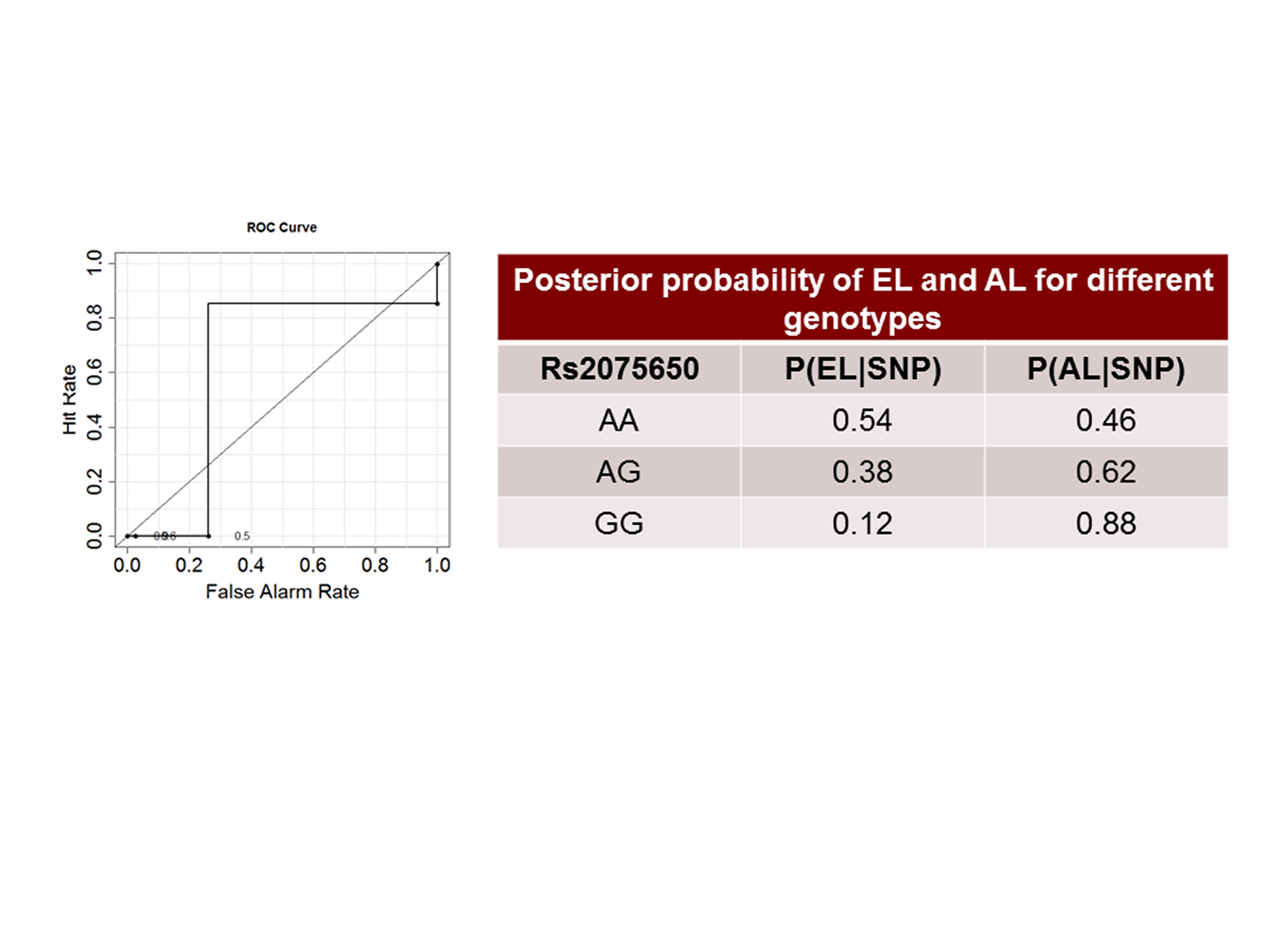

Supplement: Figure S19 — Predictive value of the SNP rs2075650 in TOMM40/APOE in the discovery set. The table reports the posterior probability of exceptional and average longevity for different genotypes of rs2075650. The ROC analysis shows that this SNP alone cannot optimize the trade off between sensitivity and specificity. The area under the curve is 0.62 compared to 0.95 when 281 SNPs are used in the model (Figure S7, top, left panel). Note that some threshold on the posterior probability can produce an accuracy that is worse than random classification. (TIF) [file pone.0029848.s019.tif]

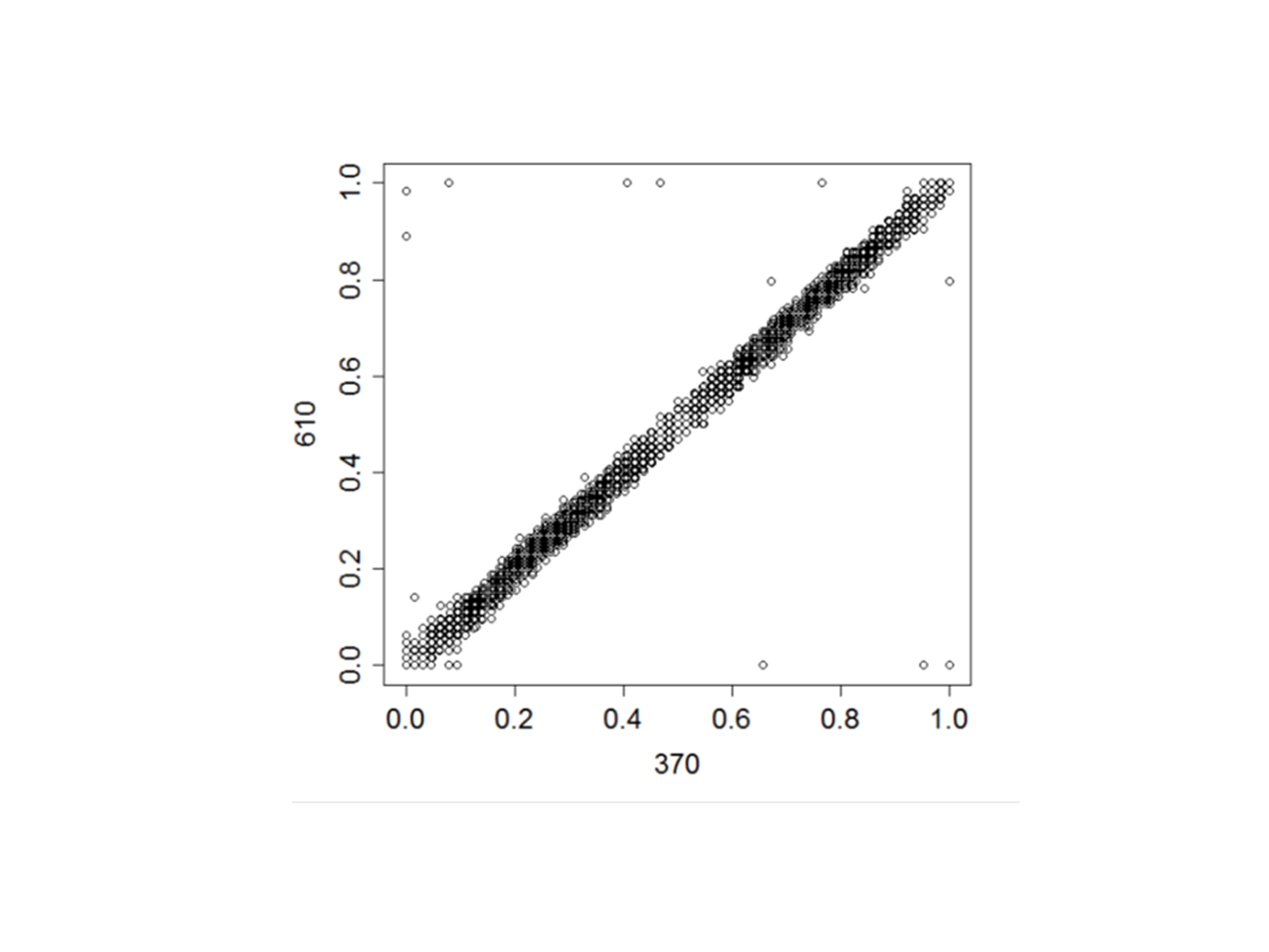

Supplement: Figure S20 — Plot of allele frequencies in 32 subjects genotyped with both the Humanhap CNV370 Illumina array (x axis) and HumanHap 610-Quad Illumina array (y-axis). Dots in the boundaries of the figure represent inconsistent SNPs between arrays. Only SNPs that had CR>97% are included. (TIF) [file pone.0029848.s020.tif]

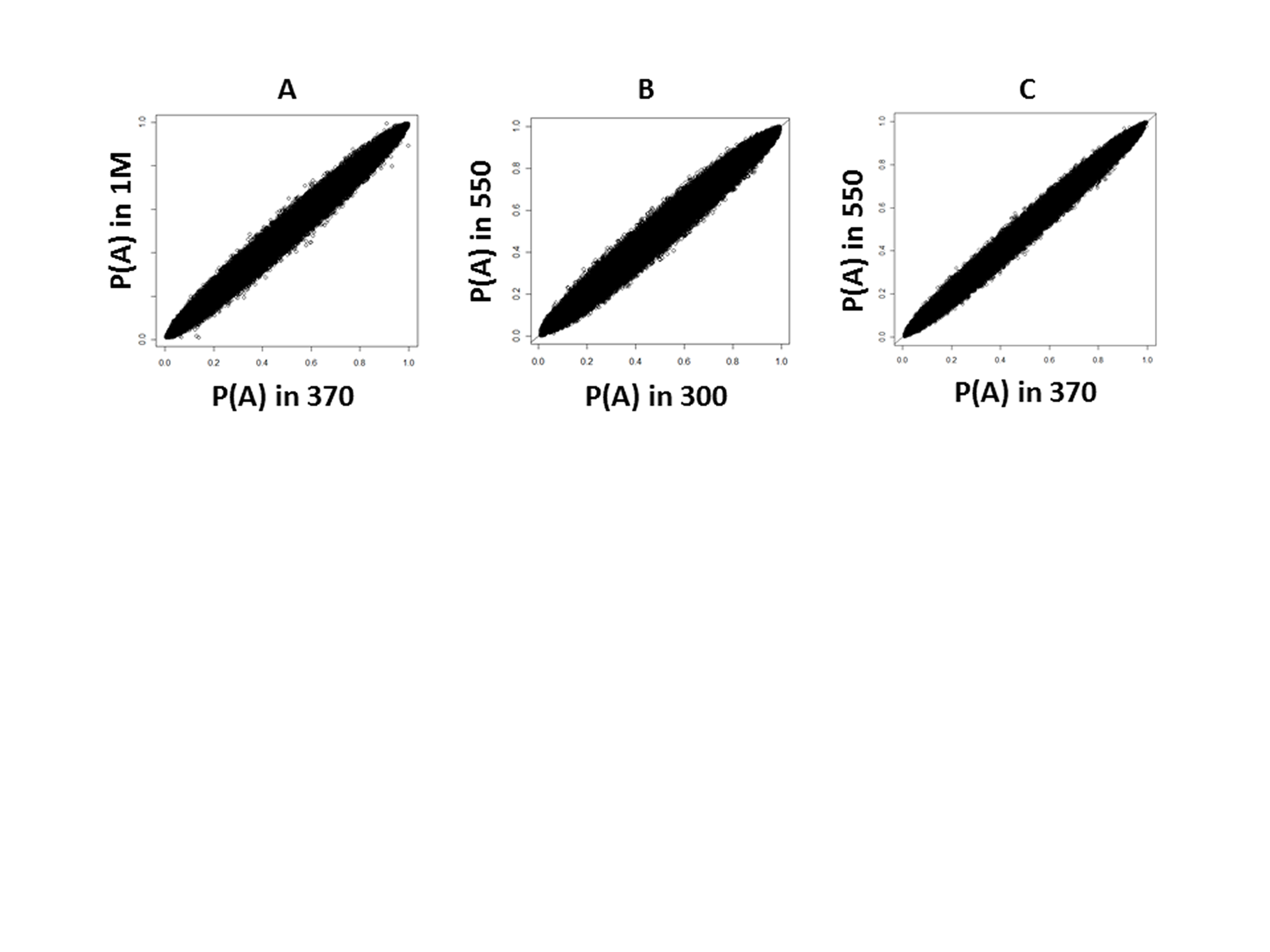

Supplement: Figure S21 — Agreement of allele frequencies in different SNP arrays. Panel A) shows the plot of allele frequencies in 573 centenarians genotyped with array HumanHap370 (x-axis) and 168 centenarians genotyped with the HumanHap 1 M (y-axis). Panel B) shows the allele frequency in 151 controls typed with array HumanHap330 (x-axis) and 863 with HumanHap 550 (y-axis). Panel C shows C) shows the allele frequency in 241 controls typed with array HumanHap370 (x-axis) and 863 with HumanHap 550 (y-axis). (TIF) [file pone.0029848.s021.tif]
