# Supplementary material for: Genetic Signatures of Exceptional Longevity in Humans
Source: PLoS One. 2012 Jan 18;7(1):e29848. doi: 10.1371/journal.pone.0029848 (PMC3261167; doi:10.1371/journal.pone.0029848)
Supplement: Table S2 — Disease prevalence in clusters of centenarians with different genetic signatures. Cardiovascular disease defined as angina, congestive heart failure, peripheral circulatory disease or myocardial infarction; pulmonary disease is asthma, chronic bronchitis or emphysema; hypertension: systolic blood pressure >140 mm Hg and/or diastolic blood pressure >90 mm Hg or on medication for HTN. (DOCX) [file pone.0029848.s023.docx]

**Supplement Table S2**

|  | **Genetic Signatures** | | | | | |  |
| --- | --- | --- | --- | --- | --- | --- | --- |
| **Disease** | **C1** | **C2** | **C3** | **C5** | **C6** | **C18** | **C26** |
| **Cancer** | **0.250** | **0.304** | **0.194** | **0.273** | **0.316** | **0.200** | **0.190** |
| **Cardio vascular disease** | **0.458** | **0.471** | **0.419** | **0.378** | **0.439** | **0.393** | **0.524** |
| **Pulmonary Disease** | **0.021** | **0.072** | **0.032** | **0.044** | **0.088** | **0.071** | **0.000** |
| **Dementia** | **0.474** | **0.451** | **0.459** | **0.532** | **0.316** | **0.214** | **0.333** |
| **Diabetes** | **0.104** | **0.014** | **0.069** | **0.067** | **0.035** | **0.143** | **0.095** |
| **Hypertension** | **0.417** | **0.435** | **0.290** | **0.311** | **0.536** | **0.357** | **0.238** |
| **Macular Degeneration** | **0.404** | **0.449** | **0.233** | **0.244** | **0.386** | **0.357** | **0.429** |
| **Stroke** | **0.104** | **0.188** | **0.097** | **0.156** | **0.158** | **0.107** | **0.286** |
