# Supplementary material for: Genetic Signatures of Exceptional Longevity in Humans
Source: PLoS One. 2012 Jan 18;7(1):e29848. doi: 10.1371/journal.pone.0029848 (PMC3261167; doi:10.1371/journal.pone.0029848)
Supplement: Table S3 — Rate of disease associated variants carried by centenarians and controls, and p-value from Student's T test. Risk alleles were derived from the GWAS catalogue at the NHGRI (downloaded in April 2011) and the Human Genome Mutation Database. The boxplots displays the rate of risk alleles carried by centenarians (blue) and controls (red). The disease described are: lupus, cholesterol level (Chol), macular degeneration (MD), Parkinson's Disease (PD), Chron's disease (chr), diabetes (diab), cardiovascular disease (CVD), cance (canc)r, Alzheimer's (AD), GWAS.pt is the group of alleles related to personality disorders that were found in GWAS, gwas.qt is the group of alleles related to QTL from GWASs and include cholesterol, BMI, obesity etc, and GWAS.cc is the group of risk alleles found from case/control GWASs so include for example cancer, PD, MD etc, cod is for coding variants from the HGMD, and all is the full set of 1214 variants. (DOCX) [file pone.0029848.s024.docx]

**Supplement Table S3**

|  | Rate in Centenarians | Rate in Controls | p-value from t-test |
| --- | --- | --- | --- |
| all | 0.401624 | 0.403561 | 2.25E-09 |
| cod | 0.311168 | 0.312752 | 0.002136 |
| gwa | 0.453007 | 0.455061 | 1.54E-06 |
| gwa.cc | 0.430155 | 0.432308 | 3.29E-05 |
| gwa.qt | 0.512866 | 0.515167 | 0.003909 |
| gwa.pt | 0.444541 | 0.444371 | 0.910248 |
| AD | 0.312661 | 0.318069 | 0.010346 |
| canc | 0.357312 | 0.36059 | 4.57E-05 |
| cvd | 0.406562 | 0.409265 | 0.050039 |
| diab | 0.420041 | 0.423085 | 0.031427 |
| chr | 0.4909 | 0.49259 | 0.398095 |
| PD | 0.447608 | 0.455504 | 0.000846 |
| MD | 0.478614 | 0.472954 | 0.163057 |
| Chol | 0.52665 | 0.529931 | 0.059284 |
| lupus | 0.385117 | 0.3819 | 0.094069 |
