# Supplementary material for: Genetic Signatures of Exceptional Longevity in Humans
Source: PLoS One. 2012 Jan 18;7(1):e29848. doi: 10.1371/journal.pone.0029848 (PMC3261167; doi:10.1371/journal.pone.0029848)
Supplement: Table S4 — List of disease associated SNPs that showed significant differences in the discovery sets. Highlighted in grey are the SNPs with risk alleles that are less common in centenarians. Some SNPs had unreported risk alleles in the original publications that are denoted with a question mark. (DOCX) [file pone.0029848.s025.docx]

**Supplement Table S4**
